# Integrative transcriptomic analysis identifies emetine as a promising candidate for overcoming acquired resistance to ALK inhibitors in lung cancer

**DOI:** 10.1002/1878-0261.13738

**Published:** 2024-11-14

**Authors:** Sang‐Min Park, Keeok Haam, Haejeong Heo, Doyeong Kim, Min‐Ju Kim, Hyo‐Jung Jung, Seongwon Cha, Mirang Kim, Haeseung Lee

**Affiliations:** ^1^ College of Pharmacy Chungnam National University Daejeon Korea; ^2^ Aging Convergence Research Center Korea Research Institute of Bioscience and Biotechnology (KRIBB) Daejeon Korea; ^3^ Personalized Genomic Medicine Research Center Korea Research Institute of Bioscience and Biotechnology (KRIBB) Daejeon Korea; ^4^ Department of Functional Genomics University of Science and Technology (UST) Daejeon Korea; ^5^ Department of Pharmacy, College of Pharmacy and Research Institute for Drug Development Pusan National University Busan Korea; ^6^ Korean Medicine (KM) Data Division Korea Institute of Oriental Medicine Daejeon Korea

**Keywords:** acquired resistance, ALK inhibitors, connectivity map, emetine, transcriptome‐based drug repositioning

## Abstract

Anaplastic lymphoma kinase (ALK; also known as ALK tyrosine kinase receptor) inhibitors (ALKi) are effective in treating lung cancer patients with chromosomal rearrangement of *ALK*. However, continuous treatment with ALKis invariably leads to acquired resistance in cancer cells. In this study, we propose an efficient strategy to suppress ALKi resistance through a meta‐analysis of transcriptome data from various cell models of acquired resistance to ALKis. We systematically identified gene signatures that consistently showed altered expression during the development of resistance and conducted computational drug screening using these signatures. We identified emetine as a promising candidate compound to inhibit the growth of ALKi‐resistant cells. We demonstrated that emetine exhibited effectiveness in inhibiting the growth of ALKi‐resistant cells, and further interpreted its impact on the resistant signatures through drug‐induced RNA‐sequencing data. Our transcriptome‐guided systematic approach paves the way for efficient drug discovery to overcome acquired resistance to cancer therapy.

## Graphical abstract

We propose an efficient strategy to suppress ALK inhibitor (ALKi) resistance. By analyzing transcriptome data, we identified emetine as a potential inhibitor. We demonstrated that emetine exhibited effectiveness in inhibiting the growth of ALKi‐resistant cells, and further interpreted its impact on the resistant signatures through drug‐induced RNA‐sequencing data.

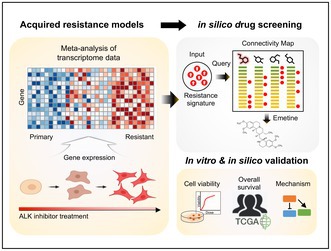

**Abbreviations**
ALK: anaplastic lymphoma kinase
ALKi: ALK inhibitor(s)
CMap: connectivity map
DEGs: differentially expressed genes
DMSO: dimethyl sulfoxide
EGFR: epidermal growth factor receptor
EML4: echinoderm microtubule‐associated protein‐like 4
EMT: epithelial‐to‐mesenchymal transition
FACS: fluorescence‐activated cell sorting
FDA: Food and Drug Administration
FDR: false discovery rate
GEO: gene expression omnibus
GSEA: gene set enrichment analysis
HDAC: histone deacetylase
HR: hazard ratio
KEGG: Kyoto Encyclopedia of Genes and Genomes
LUAD: lung adenocarcinoma
MAPK: mitogen‐activated protein kinase
MSigDB: molecular signature database
NES: normalized enrichment analysis
NSCLC: non‐small cell lung cancer
PCA: principal component analysis
PI: propidium iodide
PI3K: phosphoinositide 3‐kinase
RNA‐seq: RNA sequencing
SRP: signal recognition particle
ssGSEA: single‐sample gene set enrichment analysis
TGF‐β: transforming growth factor‐beta
TKI: tyrosine kinase inhibitor
TPM: transcripts per million

## 1 Introduction

Lung cancer is the leading cause of cancer‐related deaths worldwide, accounting for one‐fourth of all cancer‐related deaths [1]. The vast majority (~ 85%) of lung cancer patients are diagnosed with non‐small cell lung cancer (NSCLC), of which 40–50% spread beyond the lungs to other organs at the time of diagnosis [2]. In 2007, Echinoderm microtubule‐associated protein‐like 4 (*EML4*)–anaplastic lymphoma kinase (*ALK*) fusion was first detected in NSCLC [3], occurring in a subset of patients who are typically younger and non‐smokers [4]. ALK inhibitors (ALKi) have shown great promise in treating *ALK* fusion‐positive NSCLC, with crizotinib, ceritinib, and alectinib demonstrating high efficacy [5, 6, 7]. However, despite their initial success, patients inevitably develop acquired resistance to these treatments [8]. While certain mechanisms contributing to acquired resistance have been elucidated, including the emergence of secondary mutations in the *ALK* gene or activation of alternative signaling pathways that bypass the ALK‐dependent growth signals, there remain several aspects that require further investigation [9]. As this resistance is a widespread phenomenon across multiple cancer types, urgent efforts are required to develop effective strategies to overcome or prevent drug resistance in NSCLC and other cancer types [10].

The response of cancer cells to drugs is controlled by a complex biomolecular network composed of multiple interconnected signaling pathways, characterized by intricate feedback and crosstalk structures [11, 12]. Even if only a specific molecule is inhibited by targeted therapy, its effect propagates to other connected pathways, resulting in unexpected outcomes [13]. For example, treatment with a BRAF inhibitor in colorectal cancer cells induced feedback activation of epidermal growth factor receptor (EGFR), consequently reactivating BRAF and its downstream targets [14]. Treatment of NSCLC cells with EGFR inhibitors resulted in the reactivation of downstream mitogen‐activated protein kinase (MAPK) and phosphoinositide 3‐kinase (PI3K) signaling [15], bypassing of the activation of STAT3 [16], TNF [17], and calcium signaling [15], and activation of additional pathways, such as an antiviral defense pathway via interferons [18]—all of these effects contribute to cell survival and the resistance to drug treatment. In addition, the acquisition of the epithelial‐to‐mesenchymal transition (EMT) phenotype after treatment with EGFR or KRAS inhibitors conferred resistance in NSCLC cells [19, 20]. These heterogeneous and unexpected responses of cancer cells have been a hurdle in predicting and coping with drug resistance. To understand drug resistance mechanisms, systematic investigation of genome‐wide responses to drugs, beyond a single targeted pathway, is needed.

Due to recent advances in high‐throughput omics technologies, vast amounts of omics data obtained from cancer models have become publicly available. Among multiple omics datasets, the transcriptome data has been reported to be the most informative in predicting the drug response of human cancer cells [21]. Transcriptomic profiling efforts have facilitated the characterization of molecular changes during the development of cancer resistance to various anticancer agents, including ALKi [22, 23, 24, 25]. Once the gene expression signature, a set of genes involved in drug resistance, is identified, potential drugs modulating this signature at the expression level can be predicted using drug‐induced transcriptome data [e.g., the data from the Connectivity Map (CMap)] [26, 27]. The utility of this efficient approach to address drug resistance has been demonstrated in diverse cellular and *in vivo* models of cancers [22, 28, 29] and for treating non‐cancer diseases [30, 31, 32].

In this study, we systematically investigated the dynamic changes in the transcriptome of *ALK* fusion‐positive NSCLC cells in the process of acquiring resistance to ALKi. We identified common gene expression signatures conserved in resistance to three different ALKi and predicted the drugs that regulate these signature genes. The selected candidate drugs were demonstrated to be effective in inhibiting the growth of cancer cells with acquired resistance, and the clinical significance of these resistance signatures was interpreted. Our findings provide transcriptome‐based insights into novel therapeutic strategies that may overcome acquired resistance to ALKi.

## 2 Materials and methods

### 2.1 Processing of public transcriptome data

Data sets containing genome‐wide gene expression profiles of H3122 cells and ALKi‐treated H3122 cells were manually collected from the Gene Expression Omnibus (GEO) database (Accession numbers: GSE62663, GSE89127, GSE49508, and GSE81484) (Tables S1 and S2). The expression data were preprocessed dependent on the platform measuring expression values, for example, microarray or RNA sequencing (RNA‐seq). For RNA‐seq data, raw fastq files were downloaded from the European Nucleotide Archive (ENA), and their adapter sequences were trimmed using trimgalore (https://www.bioinformatics.babraham.ac.uk/). The trimmed reads were aligned to the reference genome (GRCh38) using star (v2.7.9a). The expression value per gene was estimated as a read count or the number of transcripts per kilobase million (TPM) value calculated using rsem (v1.3.3) with the human gene annotation GRCh38.104. For microarray data, normalized expression profiles were downloaded using the GEOquery package in r software (v3.6.3). Differential gene expression analysis between groups (e.g., ALKi‐resistant H3122 vs. parental H3122) was conducted using the limma package in r for microarray data or the edger package for RNA‐seq data, yielding a ranked list of genes.

### 2.2 Functional annotation using gene set enrichment analysis

Using a ranked list of genes based on differential expression, gene set enrichment analysis (GSEA) [33] was performed for the 5456 well‐curated gene sets (hallmark and canonical pathways integrating BioCarta, KEGG, PID, REACTOME, and WikiPathways) in the Molecular Signature Database (MSigDB) v7.4.1 (https://www.gsea‐msigdb.org/gsea/msigdb). GSEA quantifies the degree to which genes in a gene set are overrepresented at the top or bottom of the ranked list of genes, yielding a normalized enrichment score (NES), *P*‐value, and false discovery rate (FDR)‐adjusted *P*‐value (FDR). GSEA was performed using the fgsea package in r with parameters of a minimum size of 15, a maximum size of 500, and 10 000 permutations.

For cluster analysis of the expression profiles quantified from two different platforms, each profile of gene‐level expression was converted into a pathway level as follows. From the ranked list of genes based on differential expression in each data set (e.g., crizotinib 6 mo), we calculated the pathway activity score for 5456 gene sets, which is defined as the sign of NES × −log_10_(*P*‐value) from the GSEA. We then performed principal component analysis (PCA) using the pathway activity scores derived from each experimental condition.

### 2.3 *In silico* virtual drug screening using the connectivity map data

The CMap data [27] provide an extensive catalog of > 1.8 million transcriptomic profiles for 230 human cell lines treated with 33 609 small molecules. We downloaded the drug‐induced gene expression profiles (level 5, replicate‐collapsed *Z*‐score) from the clue.io platform, Data Library Expanded CMap LINCS Resource 2020 (CMap2020). Here, a profile is referred to as a genome‐wide vector representing differential expression levels with *Z*‐scores for 12 328 genes under unique experimental conditions (drug, cell line, treatment dose/time, and batch). Depending on the number of experiments performed per compound, multiple profiles with different cell lines or treatment doses/times can be generated for one compound. Profiles (level5_beta_trt_cp_ n720216 × 12328.gctx) were accessed using the r package, cmapr. Among a total of 1 805 898 profiles, we used only 81 907 high‐quality profiles (qc_pass ≥ 1, median_recall_rank_spearman ≤ 5, cc_q75 ≥ 0.2, pct_self_rank_q25 ≤ 5, and nsample ≥ 3) for 168 cell lines treated with 9388 compounds. To predict which compounds consistently downregulated the expression levels of the up signature genes, we scored all compounds in CMap as the following procedure. First, we calculated the Jaccard index score between the up signature genes and the 100 most‐downregulated genes obtained from each CMap *z*‐score profile. Second, the Jaccard scores of multiple CMap profiles corresponding to one compound were merged into a single score, the connectivity score. The connectivity score was defined as the negative logarithm of the hypergeometric *P*‐value for the over‐representation of the corresponding compound within the profiles that fall within the top 20% of the total Jaccard scores. Third, this procedure was repeatedly conducted with down signature genes to find compounds that consistently upregulated them. Finally, all 9388 compounds in CMap were assigned two independent scores indicating the association with the up signature or down signature genes, respectively. As a result, we focused on 23 drug candidates of which at least two of the connectivity scores have 5 or greater.

### 2.4 Survival analysis

Clinical data and gene expression profiles for lung adenocarcinoma (LUAD) patients were downloaded from The Cancer Genome Atlas (TCGA) database using the r packages tcgabiolinks (v2.31.3). A total of 516 patient datasets were utilized for this analysis. For each gene within the resistance signature, patients were stratified into two groups based on their expression levels: the upper quartile of expression values defined the high group and the lower quartile defined the low group. Kaplan–Meier analysis was then performed to test the difference in overall survival rate between the two groups by using the r package survival. Hazard ratio (HR) and *P*‐value (*P*) were computed using Cox proportional hazards regression analysis and log‐rank test, respectively. Genes with a *P*‐value lower than 0.05 were considered statistically significant in their association with overall survival.

### 2.5 Cell lines for *in vitro* validation

Human lung cancer cell lines H3122 (RRID: CVCL_5160) and H2228 (RRID: CVCL_1543), as well as their corresponding ceritinib (LDK378)‐resistant (LR) cell models, were kindly provided by B. C. Cho at Yonsei University (Seoul, Korea). LR cells were established through continuous exposure to 1 μm ceritinib for approximately 6 months, as previously described [23]. A549 (RRID: CVCL_0023) and H1793 (RRID: CVCL 1496) were purchased from ATCC (Manassas, VA, USA) and were cultured in complete DMEM medium (WelGENE, Daegu, Korea). Complete medium was supplemented with 1% antibiotics (Gibco, Thermo Fisher Scientific, Inc., Waltham, MA, USA) and 10% fetal bovine serum (WelGENE). All cells were maintained at 37 °C in a humidified atmosphere containing 5% CO_2_. All cell lines have been authenticated in the past 3 years by short tandem repeat testing and mycoplasma contamination testing.

### 2.6 Reagents

Dimethyl sulfoxide (DMSO) was purchased from Sigma‐Aldrich (St. Louis, MO, USA). Emetine (PubChem CID: 10219) was purchased from MedChemExpress (MCE, Monmouth Junction, NJ, USA).

### 2.7 Automated live‐cell analysis

Plating of 4000 H3122, H3122‐LR, H2228‐LR, or H1793 cells and 6000 A549 cells in each well of 96‐well plates (3596; Corning, Corning, NY, USA) was performed. Cells were treated with each drug and maintained at 37 °C in a humidified atmosphere containing 5% CO_2_. Photomicrographs were taken every 2 h using a Cellpro system (NanoSystem, Daejeon, Korea). Cell confluence was measured using cellpro software (NanoSystem) for up to 100 h (for H3122, H3122‐LR, H2228‐LR, and H1793 cells) or 170 h (for A549 cells) in culture.

### 2.8 RNA preparation for sequencing

Total RNA samples were isolated from H3122‐LR cells treated with vehicle (DMSO) and 10 nm emetine for 72 h using RNeasy Plus Mini Kit (QIAGEN, Hilden, Germany) according to the manufacturer's instructions including gDNA Eliminator. Purified RNA samples were quantified with NanoDrop ND‐100 spectrophotometer (Agilent, Santa Clara, CA, USA) and sample quality was estimated by the calculation of RNA Integrity Number, using Agilent 2200 TapeStation (Agilent Technologies). The RNA‐seq library was prepared using the TruSeq Stranded mRNA Kit (Illumina, San Diego, CA, USA), and sequencing was performed using the Illumina Novaseq platform to generate 150 bp paired‐end reads. The quality of sequencing reads was assessed using fastqc (v0.11.9). The Illumina adapter sequences and low‐quality base pairs were removed using trimgalore (v0.6.6). The trimmed reads were then aligned to the mouse reference genome (GRCm38) using star (v2.7.9a) with default settings. Expression levels, such as expected read count or transcript per million (TPM) per gene, were quantified using rsem (v1.3.3) along with the GRCm38.104 gene annotation. The raw fastq files and read count data are available in the GEO under accession number GSE252540.

### 2.9 Cell cycle analysis

H3122‐LR cells were collected at a population of 5 × 10^6^ cells·mL^−1^, treated with 70% ethanol, and then incubated for 1 h. Following cell washing, staining solution (300 μL) including Propidium Iodide (PI) (1.0 mg·mL^−1^) and RNase (0.5 μg·mL^−1^) were mixed, and the solution was incubated for 30 min. The flow cytometry was utilized to determine the DNA‐related PI fluorescence. The flowjo software (BD Bioscience, Franklin Lakes, NJ, USA) was used to calculate the proportions of nuclei in each cell cycle stage, including G1, S, and G2/M (FACSVerse; BD Bioscience). flowjo software was used to detect the percentage of sub‐diploid cells (apoptotic cells).

## 3 Results

### 3.1 Transcriptomic changes over time with crizotinib treatment

**Fig. 1:**
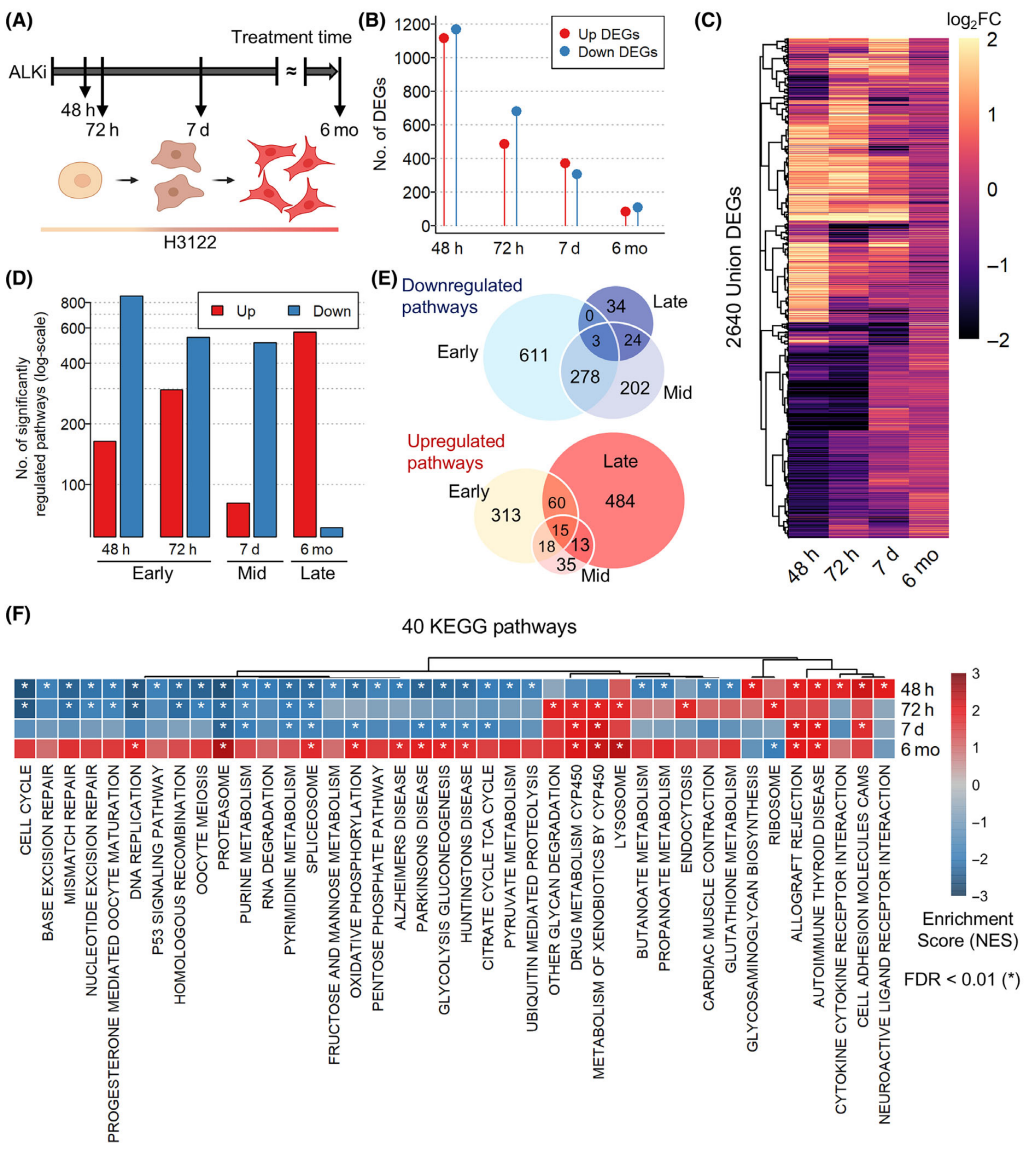
The transcriptomic dynamics in H3122 cells at different time points after crizotinib treatment. (A) A schematic representation of the time points at which transcriptome data were collected from H3122 cells following crizotinib treatment. Data were obtained at 48 h (*n* = 3), 72 h (*n* = 4), 7 days (*n* = 3) after crizotinib treatment, and at 6 months of continuous crizotinib treatment (*n* = 3). (B) The number of differentially expressed genes (DEGs) identified at each time point. (C) Expressional changes of all DEGs observed across each time point. All DEGs are the union of genes identified at each time point (Table S1). (D) The number of significantly upregulated or downregulated pathways identified at each time point following crizotinib treatment in H3122 cells. Significantly altered pathways following crizotinib treatments were obtained via conducting gene set enrichment analysis (GSEA) using the MSigDB database. (E) Venn diagrams illustrating the number of overlaps among significantly upregulated and downregulated pathways in the early, mid, and late stages. (F) GSEA results using the Kyoto Encyclopedia of Genes and Genomes (KEGG) pathway. Pathways significantly altered in the early stage were selected. ALKi, anaplastic lymphoma kinase (ALK) inhibitor; d, days; FDR, false discovery rate; h, hours; mo, months; *n*, replicate sample size; NES, normalized enrichment analysis.

Previous studies demonstrated that crizotinib treatment effectively induces cell death in *ALK* fusion‐positive lung adenocarcinoma cell lines (H3122) during the initial time points (48 and 72 h), but its efficacy diminishes due to the emergence of acquired resistance to ALKi [24, 25, 34]. We assumed that the expression of genes contributing to drug resistance would progressively increase with prolonged exposure to ALKi treatments. To explore this, we first investigated the transcriptome changes observed in H3122 cells at different time intervals following treatment with crizotinib [24, 25, 34, 35] (Table S1). Specifically, we utilized data sets collected at 48 h, 72 h, and 7 days after crizotinib treatment, as well as data obtained after 6 months of continuous crizotinib treatment (Fig. 1A). We observed that the number of differentially expressed genes (DEGs) after crizotinib treatment was higher during the initial time points, gradually decreasing as the treatment duration extended (Fig. S1A and Fig. 1B). Moreover, the expression patterns of DEGs changed temporally, where downregulated DEGs during the initial time points became upregulated at the later time point, and vice versa (Fig. 1C). To explore the biological pathways disrupted at each time point, we conducted gene set enrichment analysis (GSEA) using a comprehensive collection of well‐annotated 5456 gene sets sourced from MSigDB. Although the numbers of upregulated and downregulated DEGs were similar at each time point (Fig. 1B), GSEA results showed that the number of significantly downregulated pathways is greater than that of upregulated pathways during the initial time points (48 and 72 h) after crizotinib treatment (Fig. 1D). However, as cells developed resistance to ALKi, a distinct shift occurred, with upregulated pathways becoming increasingly predominant. Based on this observation, we divided the four‐time points into three stages: early (48 and 72 h), mid (7 days), and late (6 months). We examined the relationships among the significantly disrupted pathways at each stage and found that only a few pathways were regulated in common across all three stages (Fig. 1E). The pathways consistently upregulated at all stages included TGF‐β1 downstream targets and tumor differentiation (Fig. S1B), which is in line with the role of TGF‐β regulating cancer cell growth and resistance to multiple cancer therapies [36]. However, there were distinct differences in the pathways perturbed at each stage, as most upregulated or downregulated pathways specific to the late or the early stage, respectively (Fig. 1E). Consistent with the phenotypic outcomes that H3122 cells were initially sensitive to ALKi [24, 25, 34, 35], genes associated with cell survival, such as those involved in the cell cycle, DNA replication, nucleotide excision repair, and p53 signaling, were significantly downregulated only in the early stage, not in the late stage after acquiring resistance (Fig. 1F). These results imply that prolonged treatment with crizotinib leads to the alteration of various functional pathways beyond the initially regulated ones, indicating that cells in the late stage are in a completely different state from those in the early stage.

### 3.2 Transcriptomic changes observed in acquired resistance to ALK inhibitors

**Fig. 2:**
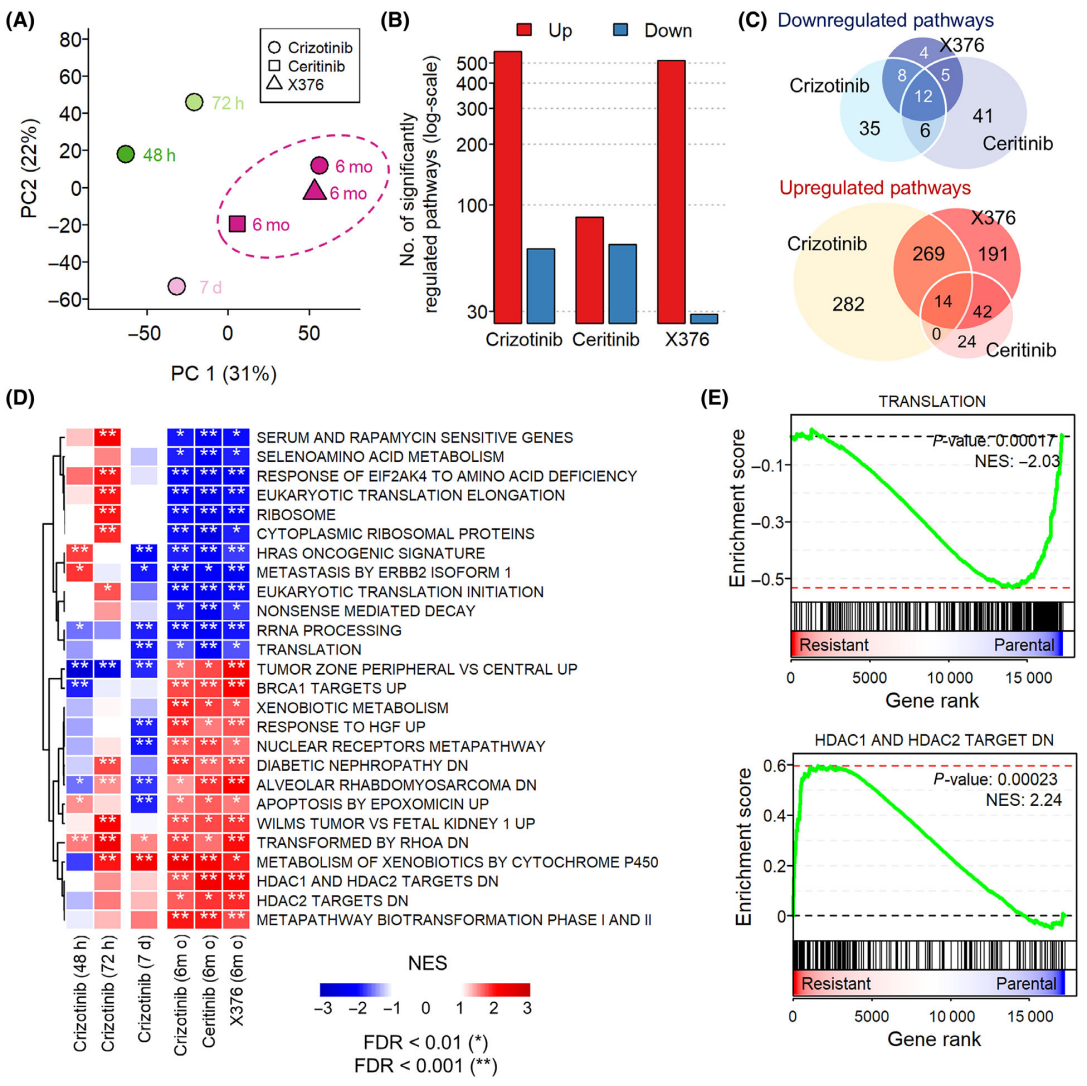
Conserved gene expression signatures of acquired resistance to ALKi. (A) Principal component analysis (PCA) illustrating distances between expression change profiles (log_2_ fold‐changes) at the indicated time points after treatment of H3122 cells with crizotinib for 48 h (*n* = 3), 72 h (*n* = 4), 7 days (*n* = 3), and 6 months (*n* = 3), as well as X‐376 (*n* = 3) and ceritinib (*n* = 2) for 6 months. (B) The number of significantly upregulated or downregulated pathways in H3122 cells that acquired resistance to the indicated drug treatment. (C) Venn diagrams showing the number of overlaps among significantly upregulated and downregulated pathways in H3122 cells that acquired resistance to the indicated drug treatment. (D) Gene set enrichment analysis (GSEA) results for the 26 pathways consistently altered in H3122 cells with acquired resistance to ALK inhibitors (ALKi). (E) GSEA plots showing the enrichment of genes related to translation and HDAC1/HDAC2 targets in an ordered list of genes based on the differential expression levels in H3122 cells with acquired resistance to ceritinib. d, days; DN, down; FDR, false discovery rate; h, hours; mo, months; *n*, replicate sample size; NES, normalized enrichment score; PC, principal component; RRNA, ribosomal RNA (rRNA).

To identify the general effects of prolonged ALK inhibition on cells, we examined whether extended treatment with other ALKi results in similar expressional changes as observed with crizotinib. We collected additional transcriptome data derived from H3122 cells that underwent continuous treatment with other ALKi, ceritinib, or X376, over a period of 6 months (Table S2) [23, 35]. Principal component analysis (PCA) based on transcriptomic changes induced by ALKi treatments showed that the early stage of crizotinib and the late stage of three ALKi were completely distant, while the late stages of the three drugs were relatively close (Fig. 2A). Similar to crizotinib, prolonged exposure to ceritinib or X376 resulted in an upregulation, rather than downregulation, of genes involved in various biological pathways (Fig. 2B,C). We assumed that the common gene expression signatures observed in resistance against these three ALKi could inform insights into the key mechanisms that must be repressed to overcome acquired resistance to ALKi. Since these transcriptome data were obtained under different experimental conditions with different experimenters and platforms measuring gene expression (Figs S1A and S2A–C), we attempted to extract conserved features from pathway‐level GSEA results that are robust to variations in heterogeneous data by considering overall changes in groups of functionally related genes, rather than individual genes. We selected 26 conserved pathways based on two criteria: (a) they were significantly altered in at least two acquired resistance cell models derived from each ALKi, and (b) they were upregulated or downregulated in common in all three resistance cell models derived from each ALKi (Fig. 2D). They consist of 14 upregulated and 12 downregulated pathways and show enrichment patterns of the late stage that are clearly distinct from those of the early and mid‐stages. Among the downregulated pathways, various processes related to translation were highly enriched, including translation initiation, ribosomal RNA processing, nonsense‐mediated decay, ribosomal proteins, and signal recognition particle (SRP)‐dependent co‐translational protein (Fig. 2D). It is probably because the neoplasm formed during the development of therapy resistance limits the supply of nutrients and oxygen, thereby reducing global protein synthesis (Fig. 2E) [37]. This neoplasm eventually leads to the selective translation of genes essential for adaptation and survival [37], and these genes will be revealed as upregulated genes or pathways in resistant populations. Notably, the target genes of histone deacetylase (HDAC) were significantly overexpressed in the cells displaying acquired resistance (Fig. 2E). This suggests that HDAC inhibition could impede ALKi resistance, which has already been demonstrated in a previous study [23]. Thus, we assumed that gene signatures in the dysregulated pathways of the late state are plausible targets that should be counter‐regulated to overcome acquired resistance to ALKi.

### 3.3 *In silico* drug screening based on resistance signatures

**Fig. 3:**
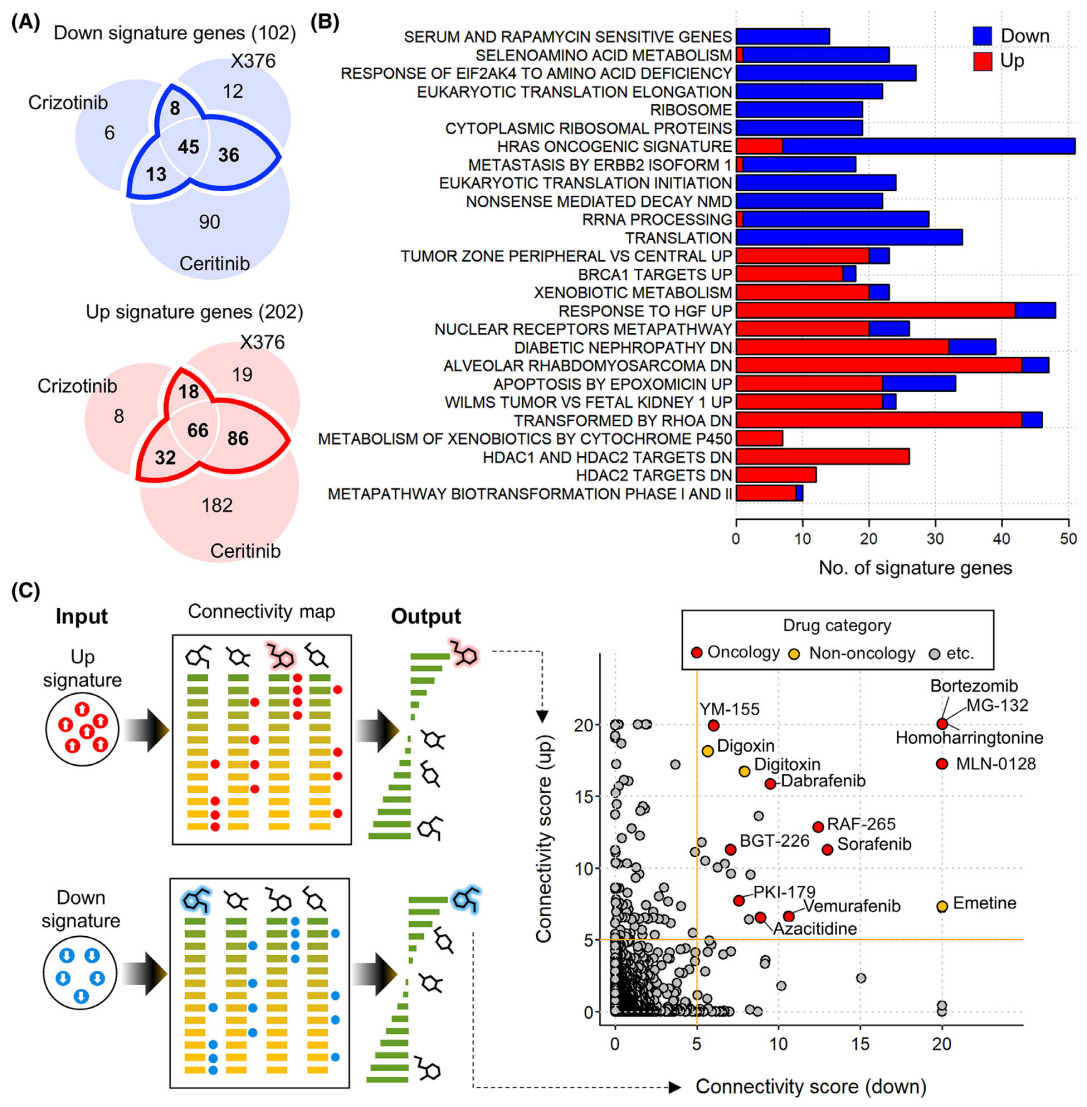
*In silico* drug screening based on resistance signatures. (A) Venn diagrams illustrating the number of overlaps among differentially expressed genes (DEGs) in resistant models induced by crizotinib (*n* = 3), X‐376 (*n* = 3), or ceritinib (*n* = 2) treatments. Numbers in parentheses indicate the count of DEGs shared by at least two of these resistance models. (B) The number of signature genes involved in each of the 26 pathways. (C) Schematic illustration of the CMap approach using up and down signatures (left) and the resulting list of top candidate drugs (right). CMap, connectivity map; DN, down; *n*, replicate sample size; RRNA, ribosomal RNA (rRNA).

Next, we sought to identify drugs capable of mitigating acquired ALKi resistance by regulating the expression of genes involved in the 26 pathways. While controlling a single gene or pathway may be a feasible approach, we presumed that modulating the entire set of genes exhibiting aberrant expression across these pathways would be more effective in suppressing acquired resistance. To accomplish this, we meticulously selected genes associated with these 26 pathways, specifically those that were significantly upregulated and downregulated in at least two acquired resistance cell models derived from each ALKi, defined as up signature genes and down signature genes, respectively (Fig. 3A). As a result, we identified 202 up and 102 down signature genes as potential therapeutic targets whose expression levels should be modulated to suppress acquired resistance (Fig. 3A). The up signature primarily comprises genes altered in 14 upregulated pathways, notably those related to the response to HGF, alveolar rhabdomyosarcoma, and RHOA targets (Fig. 3B and Fig. S3A). On the other hand, the down signature showed an even distribution of genes derived from the 12 downregulated pathways. Notably, 10 of the 12 downregulated pathways contain a substantial number of ribosomal genes in common (Fig. S3B). To further evaluate the potential clinical implications of these signature genes, we investigated their association with patient survival in lung adenocarcinoma. We analyzed gene expression data alongside overall survival information for LUAD patients within the TCGA dataset. Notably, the high expression of 26 genes from the up signature significantly correlated with poorer patient outcomes (Fig. S3C). This suggests a potential role for these signature genes in clinical settings, particularly as prognostic markers for LUAD patients.

Using these signature genes, we conducted *in silico* drug screening to find drugs that are likely to inversely regulate those genes (Fig. 3B). Briefly, we searched drug‐induced transcriptome data of ~ 40 000 small molecules from the Connectivity Map (CMap) database [27] for compounds that consistently downregulated the expression of the up signature or upregulated the expression of the down signature (Section 2). As a result, we obtained two sets of top‐scoring candidate compounds of statistical significance and focused our attention on the compounds that were common to both sets. The top candidates are enriched in approved anticancer drugs and experimental compounds with potent anticancer effects (Fig. 3C). It is noteworthy that bortezomib and MG‐132, the proteasome inhibitors, scored the highest in both up‐ and downsignature‐based methods. This aligns with previous reports showing that the combination of proteasome inhibition ALKi treatment significantly induced tumor regression in ALK‐rearranged NSCLC xenograft models [38]. In addition, protein kinase inhibitors, such as RAF‐265, sorafenib, vemurafenib, and dabrafenib exhibited notably high connectivity scores. Given the essential role of the RAS–RAF–MEK–ERK signaling in the survival of *ALK* fusion‐positive tumor cells [39], concurrently targeting ALK and RAF is anticipated to serve as an effective strategy for mitigating resistance in these cells. Collectively, our prediction presents a list of reliable candidate drugs, most of which include oncology drugs with the potential to repress acquired resistance to ALKi.

### 3.4 Emetine inhibits the growth of ALKi‐resistant cells

**Fig. 4:**
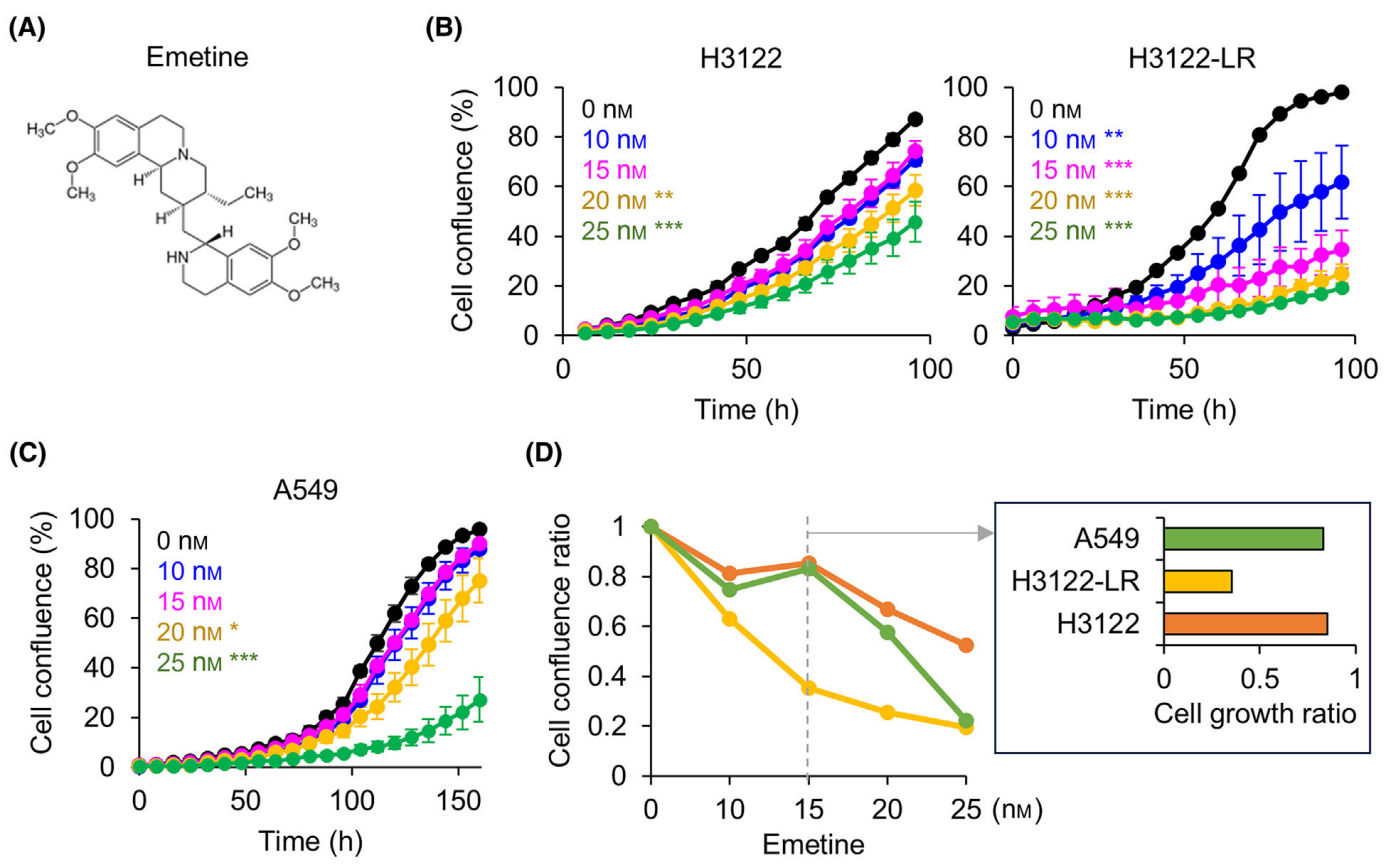
Inhibitory effects of emetine on the growth of ALK inhibitor‐resistant lung cancer cells. (A) Chemical structure of emetine. (B, C) Cell proliferation curves over time in response to indicated concentrations of emetine for human lung cancer cell lines (B) H3122, H3122‐LR, and (C) A549 cells. Error bars indicate the mean ± standard deviation based on biological replicates (*n* = 3). The statistical significance of the difference in mean values between emetine‐treated groups and the vehicle‐treated control groups was assessed using Student's *t*‐test, with significance levels denoted as *P < 0.05, **P < 0.01, and ***P < 0.005. (D) Cell confluence ratio for three cell lines, measured at 96 h following treatment with different concentrations of emetine (*n* = 3). *n*, replicate sample size.

To further explore potential therapeutic compounds that can overcome ALKi resistance, we focused on emetine, one of non‐oncology drugs among the top candidate compounds (Fig. 3C). Emetine, a bioactive alkaloid extracted from the ipecac root, has traditionally been employed in the treatment of protozoan infections (Fig. 4A). To evaluate whether emetine can induce selective inhibition of resistant cell growth, we performed live‐cell imaging analysis on ALKi‐resistant and sensitive cell lines treated with various concentrations of emetine (Fig. 4B–D). We utilized the H3122‐LR cells, a previously established ceritinib‐derived acquired resistance cell model [23], and A549 cells, representing *ALK* fusion‐negative NSCLC cells, as a control. Emetine induced a dose‐dependent inhibition of cell proliferation across all three cell lines (Fig. 4B,C). Notably, at lower concentrations (ranging from 10 to 20 nm), emetine exerted specific inhibition on H3122‐LR cells in comparison to both H3122 and A549 cells (Fig. 4D). This selective effect was further investigated using another ALKi‐resistant cell line, H2228‐LR [23]. While H2228‐LR cells also displayed dose‐dependent sensitivity to emetine, higher drug concentrations (31.25–125 nm) were required for comparable growth inhibition (Fig. S4A), suggesting potential heterogeneity in the response to emetine among different ALKi‐resistant clones.

To differentiate between acquired and intrinsic ALKi resistance, we evaluated emetine's efficacy against the *ALK* fusion‐negative NSCLC cell line H1793, which exhibited minimal response to initial crizotinib treatment (Fig. S4B) and sustained proliferation upon ceritinib exposure (Fig. S4C), indicative of intrinsic ALKi resistance. In contrast to its effect on H3122‐LR cells, emetine failed to inhibit H1793 cell growth at the tested concentrations (6.25–50 nm) (Fig. S4C). Furthermore, single‐sample gene set enrichment analysis (ssGSEA) revealed a lower expression of the up resistance signature genes in H1793, H3122, and A549 cells compared to the H3122‐LR cells (Fig. S4D). These findings collectively indicate that emetine preferentially targets NSCLC cells with acquired ALKi resistance, rather than those with intrinsic resistance.

### 3.5 Perturbing effects of emetine on the resistance signature

**Fig. 5:**
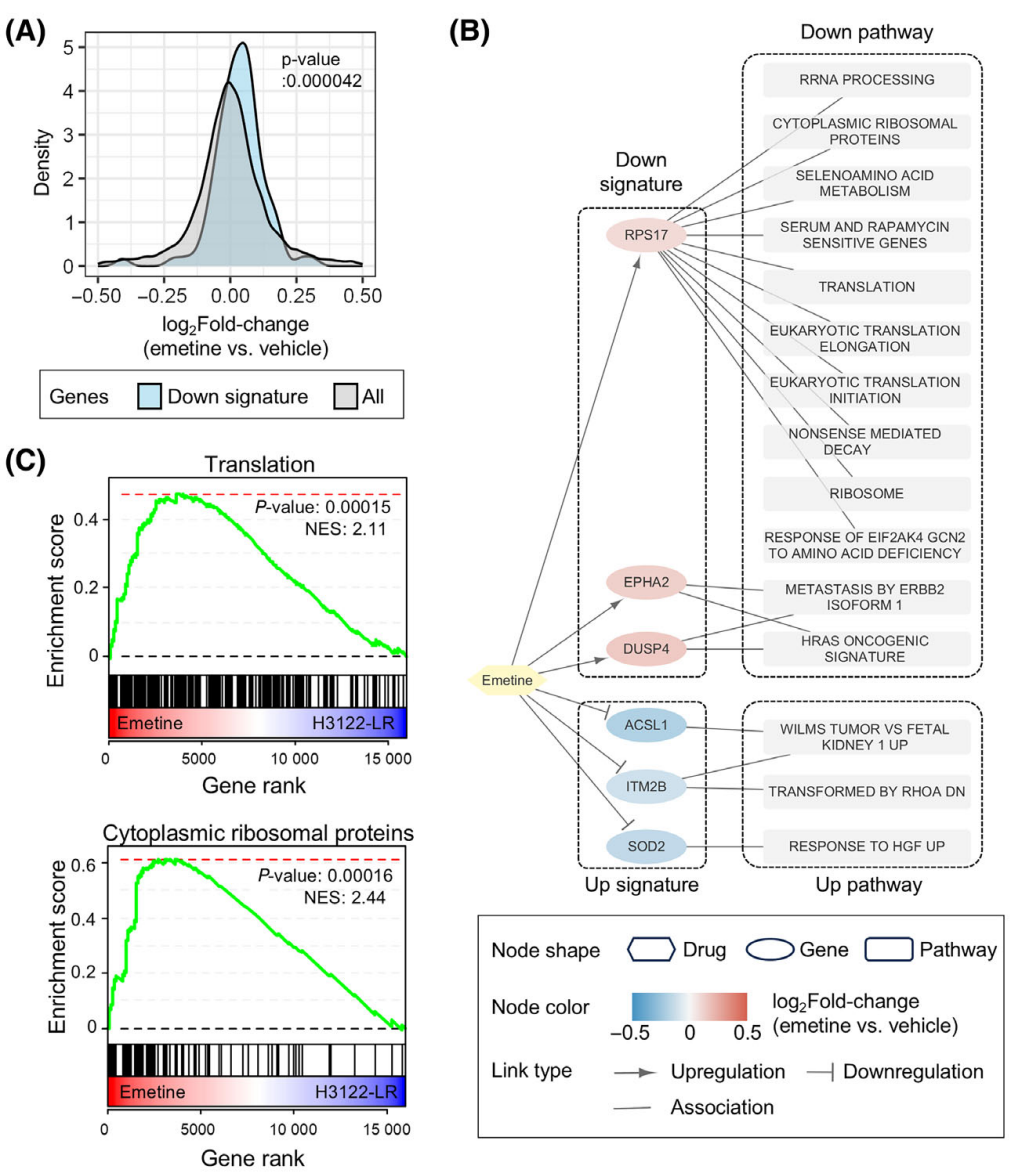
Perturbation effects of emetine on the resistance signature genes and pathways. (A) Distribution of gene expression fold‐changes in H3122 cells treated with emetine (*n* = 2). Two distinct distributions are presented: one from all genes (gray curve) and the other from the downregulated signature genes (blue curve). The statistical significance of the difference in mean log_2_ fold‐changes between these two groups was determined using Student's *t*‐test. (B) A multipartite network illustrating the perturbing effects of emetine on the ALK inhibitor (ALKi) resistance signature. Changes in gene expression (*P* < 0.05) induced by emetine treatment in H3122‐LR cells are represented by the node color in the network. The associated pathways, among the 26 identified, are linked to these genes. (C) Gene set enrichment analysis plots showing the enrichment of genes related to translation and ribosomal proteins in an ordered list of genes based on the differential expression levels in H3122‐LR cells treated with emetine. DN, down; *n*, replicate sample size; NES, normalized enrichment analysis; RRNA, ribosomal RNA (rRNA).

To evaluate whether emetine exerts its antiproliferative effects through perturbing the resistance signature, we examined transcriptomic changes in H3122‐LR cells following emetine treatment by RNA‐seq analysis (Fig. S5A). Interestingly, it induced a slight but noticeable increase in the expression of the down signature genes (Fig. 5A), while the effect on the up signature genes was minimal (Fig. S5B). Among the signature genes, emetine significantly altered six key signature genes, *RPS17*, *EPHA2*, *DUSP4*, *ASCL1*, *ITM2B*, and *SOD2* which were distinctly involved in 15 pathways out of the 26 resistance pathways identified (Fig. 5B). Interestingly, one of the down signature genes, *RPS17*, which is involved in various protein synthesis processes, was upregulated by emetine treatment. This upregulation, along with the increase in other genes related to ribosome biogenesis including translational initiation, elongation, and ribosomal proteins (Fig. 5C and Fig. S5C) implies that emetine might be compensating for the disrupted ribosomal function in H3122‐LR cells. Furthermore, the alterations in the expression of the *EPHA2*, *DUSP4*, *ASCL1*, *ITM2B*, and *SOD2*, which are implicated in several oncogenic signaling pathways, indicate a broader impact of emetine on cellular processes beyond protein synthesis.

Beyond its effects on the resistance signature, emetine downregulated genes linked to the G1‐S phase transition in H3122‐LR cells (Fig. S5D). To examine whether emetine‐induced cell growth inhibition is associated with cell cycle arrest, we conducted flow cytometry analysis. While high emetine concentrations led a slight cell cycle arrest, as evidenced by an elevated G0/G1 phase proportion (Fig. S5E), lower concentrations did not induce significant cell cycle perturbations. These findings suggest that emetine's primary antiproliferative mechanism in H3122‐LR cells at lower doses may be independent of cell cycle modulation, although further investigation is required to fully elucidate the underlying processes.

## 4 Discussion

This study presents a viable strategy for identifying drug candidates to overcome resistance in cancers by leveraging the unique transcriptomic signatures of cancer cells that have acquired resistance to specific drugs. This approach has the potential to address the limitations of current methods, which often focus on targeting single driver mutation and can be ineffective against complex resistance mechanisms. We focused on ALKi‐resistant lung adenocarcinoma cells (H3122‐LR) as a model system. We observed that transcriptomic changes in H3122‐LR compared to parental cells were distinct from those seen in H3122 cells initially exposed to ALKi. Notably, no secondary mutations in the ALK tyrosine kinase domain were detected in H3122‐LR cells [23], suggesting that this resistance is independent of ALK. We hypothesized that potential therapeutic targets for overcoming resistance would be reflected in the transcriptomic signatures of resistant cells. We compared the transcriptomes of H3122‐LR cells with three other independent ALKi‐resistant models and identified 26 commonly altered pathways including, targets of HDAC, xenobiotic metabolic process, and translational processes. From these pathways, we selected a set of signature genes that were consistently dysregulated across all models. We reasoned that targeting these signature genes as a whole, rather than individual genes, might be more effective in overcoming resistance, as it is likely to disrupt the complex network of pathways involved [40]. To assess this hypothesis, we employed the CMap approach, a powerful tool for transcriptomic signature‐based drug screening. This approach led to the identification of emetine, a potent and selective inhibitor of H3122‐LR cell growth at low concentrations. This finding supports our hypothesis that targeting the entire signature of resistance‐associated genes, rather than a single gene, may be an effective strategy to overcome acquired resistance, which often involves complex transcriptional changes across multiple pathways.

A key strength of the CMap approach lies in its reliance solely on transcriptomic signatures associated with distinct cellular states. This makes CMap a highly versatile tool for drug screening across a wide range of diseases involving transcriptomic changes [32, 41]. Examples include the identification of atorvastatin, a cholesterol‐lowering drug, as a potential candidate to reverse chemotherapeutic resistance in cancer cells [22] and bortezomib as a potent inhibitor of metastasis in lung adenocarcinoma [29]. Additionally, CMap has shown promise beyond cancer, with studies identifying AZ‐628 as a potent RIP3 inhibitor for halting osteoarthritis progression [30]. The successful application of CMap in our study for ALKi‐resistant cell models suggests its broader applicability to tackle resistance problems in other cancers driven by different actionable mutations. EGFR‐mutant lung cancer, for instance, also undergoes significant transcriptomic changes during the development of resistance to EGFR inhibitors [42], making them potential targets for CMap‐driven drug discovery.

In contrast to our signature‐based approach, previous studies [12, 43, 44, 45] utilized a model‐driven approach using a mathematical model to simulate the effects of drug perturbations on feedback activity in a cancer signaling network. To counteract adaptive resistance to MAPK and PI3K pathway inhibitors, their study pinpointed Src as a critical combinatorial target [12]. Treatments with these inhibitors activate Src, which then interferes with the targeted drug pathways, contributes to cell survival through multiple bypass signaling pathways, and consequently reduces drug efficacy. However, focusing on a single molecule such as Src could be insufficient, as this leaves out other potentially influential molecules within the intricate resistance mechanisms. Our data‐driven approach, focusing on the genome‐wide transcriptomic signature of resistance, may complement these limitations. Integrating the strengths of data‐ and model‐driven approaches could pave the way for the development of hybrid computational models that integrate transcriptomic data with network simulations to predict and counteract resistance mechanisms with actionable insights.

Emetine is a well‐known protein synthesis inhibitor commonly used as an emetic and antiparasitic drug. Previous studies demonstrate its diverse effects on cancer cells, including inhibiting NSCLC migration and invasion by regulating MAPK signaling pathways [46], synergistically enhancing cisplatin efficacy in NSCLC [47], and overcoming EGFR‐TKI resistance by blocking heat shock factor 1 (HSF1) activity [48]. These findings collectively suggest the promising clinical applicability of emetine in treating ALKi‐resistant NSCLC, as well as parental NSCLC. To gain molecular insights into emetine's selective cytotoxicity towards H3122‐LR cells, we investigated transcriptomic alterations within the resistant signatures after emetine treatment. Our multipartite perturbation network analysis revealed expression changes in six signature genes across 15 distinct pathways. This network‐based approach revealed that emetine's influence on ALKi resistance is not merely a matter of modulating individual genes but involves a network of interrelated pathways.

However, several limitations of this study require consideration. Firstly, while emetine's antiproliferative effects are evident, the precise molecular mechanisms remain to be elucidated. This study primarily focuses on establishing an efficient transcriptomic signature‐based drug repositioning strategy, and a detailed mechanistic exploration of emetine was beyond its scope. Secondly, the identified resistance signatures obtained from H3122 cells were primarily associated with ALKi resistance but did not directly pinpoint the underlying mechanisms for acquiring resistance. Further investigation is required to fully understand the molecular mechanisms involved in developing this resistance. Thirdly, our findings were based on a limited number of cell lines. Additional experiments, including *in vivo* studies and clinical trials, are necessary to evaluate the efficacy and safety of emetine and to assess the generalizability of our approach. Finally, our approach may not be universally applicable to all forms of drug resistance. Despite these limitations, our study provides a proof‐of‐concept for an efficient drug discovery strategy by leveraging the power of transcriptomics and computational analysis.

## 5 Conclusion

This study presents an efficient transcriptome‐guided drug repositioning approach for identifying potential candidates against acquired resistance to targeted therapies. By analyzing the distinct gene expression signatures of ALKi‐resistant lung cancer cells and integrating them with CMap data, we identified emetine as a promising candidate for overcoming ALKi resistance. Through emetine‐induced transcriptome analysis, we obtained mechanistic insights into how emetine selectively targets ALKi‐resistant cells. Considering the ever‐growing volume of cancer genomic and chemical genomic data, our study presents an efficient strategy for accelerating the discovery of novel therapeutic options in the fight against drug resistance.

## Conflict of interest

The authors declare no conflict of interest.

## Author contributions

HL led the overall study, performed the *in silico* analysis, and wrote the manuscript. S‐MP conceived the study design, performed the *in silico* analysis, and wrote the manuscript. MK led the *in vitro* experiments and wrote the manuscript. KH, HH, and H‐JJ conducted *in vitro* experiments. DK and M‐JK analyzed RNA‐seq data. SC discussed and interpreted the data. All authors contributed to the writing and revision of the manuscript and endorsed the final version.

### Peer review

The peer review history for this article is available at https://www.webofscience.com/api/gateway/wos/peer‐review/10.1002/1878‐0261.13738.

## Supporting information


**Fig. S1.** Transcriptomic responses of H3122 cells to crizotinib treatment.
**Fig. S2.** Transcriptomic responses of H3122 cells to ALK inhibitor (ALKi) treatment.
**Fig. S3.** Resistance signature genes for ALK inhibitor.
**Fig. S4.** Effect of emetine on H2228‐LR and H1793 cells.
**Fig. S5.** Transcriptomic responses of H3122‐LR cells to emetine treatment.

**Table S1.** Public transcriptome datasets derived from H3122 cell lines with different durations of crizotinib treatment.
**Table S2.** Public transcriptome datasets derived from H3122 cell lines with ceritinib or X376 treatment.

## Data Availability

Publicly available transcriptome datasets for H3122 cells were retrieved from GEO database (GSE62663, GSE89127, GSE49508, and GSE81484). A portion of the data used for this study was obtained from the Genome‐InfraNet (IDs: 1711048605, 1711048587, 1711041874, 1711041928, and 1711072542) of the Korea Bioinformation Center. Functional annotations for the analyzed genes were retrieved from the MsigDB (https://www.gsea‐msigdb.org/gsea/msigdb). RNA‐sequencing data generated in this study for H3122‐LR cells following emetine treatment were deposited in the GEO database under accession number GSE252540. The raw RNA‐seq data were also deposited in K‐BDS (Korea BioData Station, https://kbds.re.kr) under accession number KAP240831.

## References

[1] Siegel RL , Miller KD , Fuchs HE , Jemal A . Cancer statistics, 2021. CA Cancer J Clin. 2021;71(1):7–33. 33433946 10.3322/caac.21654

[2] Tamura T , Kurishima K , Nakazawa K , Kagohashi K , Ishikawa H , Satoh H , et al. Specific organ metastases and survival in metastatic non‐small‐cell lung cancer. Mol Clin Oncol. 2015;3(1):217–221. 25469298 10.3892/mco.2014.410 PMC4251107

[3] Soda M , Choi YL , Enomoto M , Takada S , Yamashita Y , Ishikawa S , et al. Identification of the transforming EML4‐ALK fusion gene in non‐small‐cell lung cancer. Nature. 2007;448(7153):561–566. 17625570 10.1038/nature05945

[4] Shaw AT , Yeap BY , Mino‐Kenudson M , Digumarthy SR , Costa DB , Heist RS , et al. Clinical features and outcome of patients with non‐small‐cell lung cancer who harbor EML4‐ALK. J Clin Oncol. 2009;27(26):4247–4253. 19667264 10.1200/JCO.2009.22.6993 PMC2744268

[5] Shaw AT , Kim D‐W , Nakagawa K , Seto T , Crinó L , Ahn M‐J , et al. Crizotinib versus chemotherapy in advanced ALK‐positive lung cancer. N Engl J Med. 2013;368(25):2385–2394. 23724913 10.1056/NEJMoa1214886

[6] Shaw AT , Kim D‐W , Mehra R , Tan DSW , Felip E , Chow LQM , et al. Ceritinib in ALK‐rearranged non–small‐cell lung cancer. N Engl J Med. 2014;370(13):1189–1197. 24670165 10.1056/NEJMoa1311107 PMC4079055

[7] Ou SHI , Ahn JS , De Petris L , Govindan R , Yang JCH , Hughes B , et al. Alectinib in crizotinib‐refractory alk‐rearranged non‐small‐cell lung cancer: a phase II global study. J Clin Oncol. 2016;34(7):661–668. 26598747 10.1200/jco.2015.63.9443

[8] Shaw AT , Gandhi L , Gadgeel S , Riely GJ , Cetnar J , West H , et al. Alectinib in ALK‐positive, crizotinib‐resistant, non‐small‐cell lung cancer: a single‐group, multicentre, phase 2 trial. Lancet Oncol. 2016;17(2):234–242. 26708155 10.1016/S1470-2045(15)00488-X PMC4752892

[9] Della Corte CM , Viscardi G , Di Liello R , Fasano M , Martinelli E , Troiani T , et al. Role and targeting of anaplastic lymphoma kinase in cancer. Mol Cancer. 2018;17(1):30. 29455642 10.1186/s12943-018-0776-2 PMC5817803

[10] Amirouchene‐Angelozzi N , Swanton C , Bardelli A . Tumor evolution as a therapeutic target. Cancer Discov. 2017;7(8):805–817. 10.1158/2159-8290.CD-17-0343 28729406

[11] Sun C , Bernards R . Feedback and redundancy in receptor tyrosine kinase signaling: relevance to cancer therapies. Trends Biochem Sci. 2014;39(10):465–474. 25239057 10.1016/j.tibs.2014.08.010

[12] Park SM , Hwang CY , Choi J , Joung CY , Cho KH . Feedback analysis identifies a combination target for overcoming adaptive resistance to targeted cancer therapy. Oncogene. 2020;39(19):3803–3820. 32157217 10.1038/s41388-020-1255-y

[13] Chen SH , Lahav G . Two is better than one; toward a rational design of combinatorial therapy. Curr Opin Struct Biol. 2016;41:145–150. 27521655 10.1016/j.sbi.2016.07.020 PMC5469547

[14] Corcoran RB , Ebi H , Turke AB , Coffee EM , Nishino M , Cogdill AP , et al. EGFR‐mediated reactivation of MAPK signaling contributes to insensitivity of BRAF‐mutant colorectal cancers to RAF inhibition with vemurafenib. Cancer Discov. 2012;2(3):227–235. 22448344 10.1158/2159-8290.CD-11-0341 PMC3308191

[15] Mulder C , Prust N , Van Doorn S , Reinecke M , Kuster B , Van Bergen En Henegouwen P , et al. Adaptive resistance to EGFR‐targeted therapy by calcium signaling in NSCLC cells. Mol Cancer Res. 2018;16(11):1773–1784. 29967110 10.1158/1541-7786.MCR-18-0212

[16] Lee HJ , Zhuang G , Cao Y , Du P , Kim HJ , Settleman J . Drug resistance via feedback activation of Stat3 in oncogene‐addicted cancer cells. Cancer Cell. 2014;26(2):207–221. 25065853 10.1016/j.ccr.2014.05.019

[17] Gong K , Guo G , Gerber DE , Gao B , Peyton M , Huang C , et al. TNF‐driven adaptive response mediates resistance to EGFR inhibition in lung cancer. J Clin Invest. 2018;128(6):2500–2518. 29613856 10.1172/JCI96148 PMC5983340

[18] Gong K , Guo G , Panchani N , Bender ME , Gerber DE , Minna JD , et al. EGFR inhibition triggers an adaptive response by co‐opting antiviral signaling pathways in lung cancer. Nat Cancer. 2020;1(4):394–409. 33269343 10.1038/s43018-020-0048-0 PMC7706867

[19] Adachi Y , Ito K , Hayashi Y , Kimura R , Tan TZ , Yamaguchi R , et al. Epithelial‐to‐mesenchymal transition is a cause of both intrinsic and acquired resistance to KRAS G12C inhibitor in KRAS G12C‐mutant non‐small cell lung cancer. Clin Cancer Res. 2020;26(22):5962–5973. 32900796 10.1158/1078-0432.CCR-20-2077

[20] Sato H , Yamamoto H , Sakaguchi M , Shien K , Tomida S , Shien T , et al. Combined inhibition of MEK and PI3K pathways overcomes acquired resistance to EGFR‐TKIs in non‐small cell lung cancer. Cancer Sci. 2018;109(10):3183–3196. 30098066 10.1111/cas.13763 PMC6172047

[21] Corsello SM , Nagari RT , Spangler RD , Rossen J , Kocak M , Bryan JG , et al. Discovering the anticancer potential of non‐oncology drugs by systematic viability profiling. Nat Cancer. 2020;1(2):235–248. 32613204 10.1038/s43018-019-0018-6 PMC7328899

[22] Hong S‐K , Lee H , Kwon O‐S , Song N‐Y , Lee H‐J , Kang S , et al. Large‐scale pharmacogenomics based drug discovery for ITGB3 dependent chemoresistance in mesenchymal lung cancer. Mol Cancer. 2018;17:175. 30563517 10.1186/s12943-018-0924-8 PMC6299529

[23] Yun MR , Lim SM , Kim SK , Choi HM , Pyo KH , Kim SK , et al. Enhancer remodeling and microRNA alterations are associated with acquired resistance to ALK inhibitors. Cancer Res. 2018;78(12):3350–3362. 29669761 10.1158/0008-5472.CAN-17-3146

[24] Yun MR , Choi HM , Lee YW , Joo HS , Park CW , Choi JW , et al. Targeting YAP to overcome acquired resistance to ALK inhibitors in ALK‐rearranged lung cancer. EMBO Mol Med. 2019;11(12):1–17. 10.15252/emmm.201910581 PMC6895608 31633304

[25] Rusan M , Li K , Li Y , Christensen CL , Abraham BJ , Kwiatkowski N , et al. Suppression of adaptive responses to targeted cancer therapy by transcriptional repression. Cancer Discov. 2018;8(1):59–73. 29054992 10.1158/2159-8290.CD-17-0461 PMC5819998

[26] Lamb J , Crawford ED , Peck D , Modell JW , Blat IC , Wrobel MJ , et al. The connectivity map: using. Science. 2006;313:1929–1935. 17008526 10.1126/science.1132939

[27] Subramanian A , Narayan R , Corsello SM , Peck DD , Natoli TE , Lu X , et al. A next generation connectivity map: L1000 platform and the first 1,000,000 profiles. Cell. 2017;171(6):1437–1452.e17. 29195078 10.1016/j.cell.2017.10.049 PMC5990023

[28] Lee H , Kang S , Kim W . Drug repositioning for cancer therapy based on large‐scale drug‐induced transcriptional signatures. PLoS One. 2016;11(3):e0150460. 26954019 10.1371/journal.pone.0150460 PMC4783079

[29] Kwon OS , Lee H , Kong HJ , Kwon EJ , Park JE , Lee W , et al. Connectivity map‐based drug repositioning of bortezomib to reverse the metastatic effect of GALNT14 in lung cancer. Oncogene. 2020;39(23):4567–4580. 32388539 10.1038/s41388-020-1316-2

[30] Jeon J , Noh H‐J , Lee H , Park H‐H , Ha Y‐J , Park SH , et al. TRIM24‐RIP3 axis perturbation accelerates osteoarthritis pathogenesis. Ann Rheum Dis. 2020;79:1635–1643. 32895234 10.1136/annrheumdis-2020-217904 PMC7677493

[31] Brum AM , van de Peppel J , Nguyen L , Aliev A , Schreuders‐Koedam M , Gajadien T , et al. Using the connectivity map to discover compounds influencing human osteoblast differentiation. J Cell Physiol. 2018;233(6):4895–4906. 29194609 10.1002/jcp.26298

[32] Kwon OS , Kim W , Cha HJ , Lee H . In silico drug repositioning: from large‐scale transcriptome data to therapeutics. Arch Pharm Res. 2019;42(10):879–889. 31482491 10.1007/s12272-019-01176-3

[33] Subramanian A , Tamayo P , Mootha VK , Mukherjee S , Ebert BL , Gillette MA , et al. Gene set enrichment analysis: a knowledge‐based approach for interpreting genome‐wide expression profiles. Proc Natl Acad Sci USA. 2005;102(43):15545–15550. 16199517 10.1073/pnas.0506580102 PMC1239896

[34] Caffa I , D'Agostino V , Damonte P , Soncini D , Cea M , Monacelli F , et al. Fasting potentiates the anticancer activity of tyrosine kinase inhibitors by strengthening MAPK signaling inhibition. Oncotarget. 2015;6(14):11820–11832. 25909220 10.18632/oncotarget.3689 PMC4494907

[35] Lovly CM , McDonald NT , Chen H , Ortiz‐Cuaran S , Heukamp LC , Yan Y , et al. Rationale for co‐targeting IGF‐1R and ALK in ALK fusion‐positive lung cancer. Nat Med. 2014;20(9):1027–1034. 25173427 10.1038/nm.3667 PMC4159407

[36] Zhang M , Zhang YY , Chen Y , Wang J , Wang Q , Lu H . TGF‐β signaling and resistance to cancer therapy. Front Cell Dev Biol. 2021;9:786728. 34917620 10.3389/fcell.2021.786728 PMC8669610

[37] Lee LJ , Papadopoli D , Jewer M , del Rincon S , Topisirovic I , Lawrence MG , et al. Cancer plasticity: the role of mRNA translation. Trends Cancer. 2021;7(2):134–145. 33067172 10.1016/j.trecan.2020.09.005 PMC8023421

[38] Tanimoto A , Matsumoto S , Takeuchi S , Arai S , Fukuda K , Nishiyama A , et al. Proteasome inhibition overcomes ALK‐TKI resistance in ALK‐rearranged/TP53‐mutant NSCLC via noxa expression. Clin Cancer Res. 2021;27(5):1410–1420. 33310890 10.1158/1078-0432.CCR-20-2853

[39] Hrustanovic G , Bivona TG . RAS‐MAPK in ALK targeted therapy resistance. Cell Cycle. 2015;14(23):3661–3662. 26654768 10.1080/15384101.2015.1096103 PMC4825705

[40] Chen B , Garmire L , Calvisi DF , Chua MS , Kelley RK , Chen X . Harnessing big ‘omics’ data and AI for drug discovery in hepatocellular carcinoma. Nat Rev Gastroenterol Hepatol. 2020;17(4):238–251. 31900465 10.1038/s41575-019-0240-9 PMC7401304

[41] Musa A , Ghoraie LS , Zhang SD , Glazko G , Yli‐Harja O , Dehmer M , et al. A review of connectivity map and computational approaches in pharmacogenomics. Brief Bioinform. 2018;19(3):506–523. 28069634 10.1093/bib/bbw112 PMC5952941

[42] Kwon EJ , Cha HJ , Lee H . Systematic omics analysis identifies CCR6 as a therapeutic target to overcome cancer resistance to EGFR inhibitors. iScience. 2024;27(4):109448. 38551001 10.1016/j.isci.2024.109448 PMC10972824

[43] Choi M , Park SM , Cho KH . Evaluating a therapeutic window for precision medicine by integrating genomic profiles and p53 network dynamics. Commun Biol. 2022;5(1):924. 36071176 10.1038/s42003-022-03872-1 PMC9452682

[44] Park S , Hwang CY , Cho S , Lee D , Gong J , Lee S , et al. Systems analysis identifies potential target genes to overcome cetuximab resistance in colorectal cancer cells. FEBS J. 2019;286(7):1305–1318. 30719834 10.1111/febs.14773

[45] Hwang CY , Yu SJ , Won J‐K , Park S‐M , Noh H , Lee S , et al. Systems analysis identifies endothelin 1 axis blockade for enhancing the anti‐tumor effect of multikinase inhibitor. Cancer Gene Ther. 2021;29(6):845–858. 34363028 10.1038/s41417-021-00373-x

[46] Kim JH , Cho EB , Lee J , Jung O , Ryu BJ , Kim SH , et al. Emetine inhibits migration and invasion of human non‐small‐cell lung cancer cells via regulation of ERK and p38 signaling pathways. Chem Biol Interact. 2015;242:25–33. 26332055 10.1016/j.cbi.2015.08.014

[47] Wu TH , Chang SY , Shih YL , Huang TW , Chang H , Lin YW . Emetine synergizes with cisplatin to enhance anti‐cancer efficacy against lung cancer cells. Int J Mol Sci. 2019;20(23):5914. 31775307 10.3390/ijms20235914 PMC6928603

[48] Lee S , Jung J , Lee YJ , Kim SK , Kim JA , Kim BK , et al. Targeting HSF1 as a therapeutic strategy for multiple mechanisms of EGFR inhibitor resistance in EGFR mutant non‐small‐cell lung cancer. Cancers. 2021;13(12):2987. 34203709 10.3390/cancers13122987 PMC8232331

