# Supplementary material for: Integrative transcriptomic analysis identifies emetine as a promising candidate for overcoming acquired resistance to ALK inhibitors in lung cancer
Source: Mol Oncol. 2024 Nov 14;19(4):1155–69. doi: 10.1002/1878-0261.13738 (PMC11977641; doi:10.1002/1878-0261.13738)
Supplement: Supplementary file 1 — Fig. S1. Transcriptomic responses of H3122 cells to crizotinib treatment. Fig. S2. Transcriptomic responses of H3122 cells to ALK inhibitor (ALKi) treatment. Fig. S3. Resistance signature genes for ALK inhibitor. Fig. S4. Effect of emetine on H2228‐LR and H1793 cells. Fig. S5. Transcriptomic responses of H3122‐LR cells to emetine treatment. [file MOL2-19-1155-s002.pptx]

## Slide 1
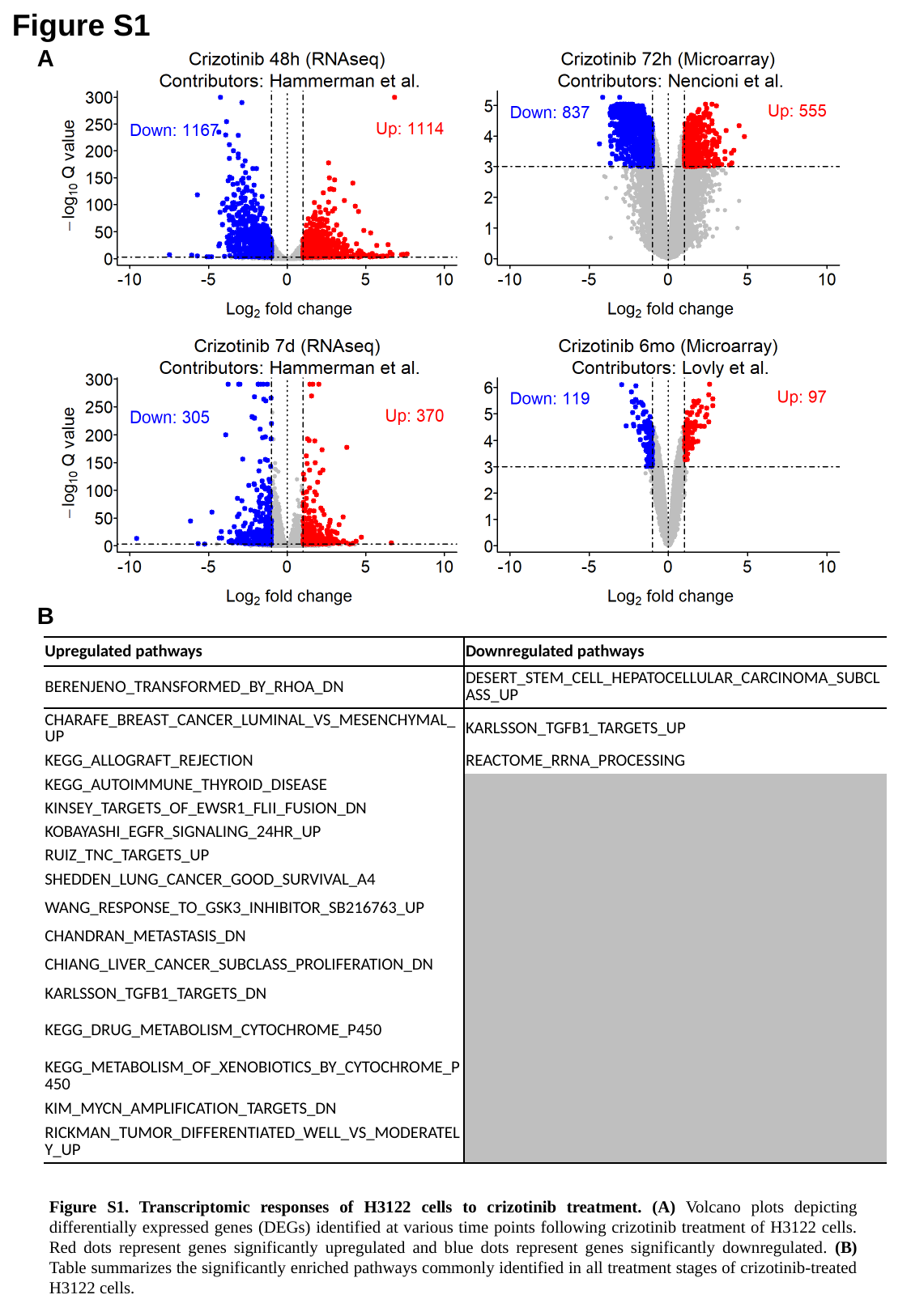

Figure S1
A
B
| Upregulated pathways | Downregulated pathways |
| --- | --- |
| BERENJENO\_TRANSFORMED\_BY\_RHOA\_DN | DESERT\_STEM\_CELL\_HEPATOCELLULAR\_CARCINOMA\_SUBCLASS\_UP |
| CHARAFE\_BREAST\_CANCER\_LUMINAL\_VS\_MESENCHYMAL\_UP | KARLSSON\_TGFB1\_TARGETS\_UP |
| KEGG\_ALLOGRAFT\_REJECTION | REACTOME\_RRNA\_PROCESSING |
| KEGG\_AUTOIMMUNE\_THYROID\_DISEASE | |
| KINSEY\_TARGETS\_OF\_EWSR1\_FLII\_FUSION\_DN | |
| KOBAYASHI\_EGFR\_SIGNALING\_24HR\_UP | |
| RUIZ\_TNC\_TARGETS\_UP | |
| SHEDDEN\_LUNG\_CANCER\_GOOD\_SURVIVAL\_A4 | |
| WANG\_RESPONSE\_TO\_GSK3\_INHIBITOR\_SB216763\_UP | |
| CHANDRAN\_METASTASIS\_DN | |
| CHIANG\_LIVER\_CANCER\_SUBCLASS\_PROLIFERATION\_DN | |
| KARLSSON\_TGFB1\_TARGETS\_DN | |
| KEGG\_DRUG\_METABOLISM\_CYTOCHROME\_P450 | |
| KEGG\_METABOLISM\_OF\_XENOBIOTICS\_BY\_CYTOCHROME\_P450 | |
| KIM\_MYCN\_AMPLIFICATION\_TARGETS\_DN | |
| RICKMAN\_TUMOR\_DIFFERENTIATED\_WELL\_VS\_MODERATELY\_UP | |
Figure S1. Transcriptomic responses of H3122 cells to crizotinib treatment. (A) Volcano plots depicting differentially expressed genes (DEGs) identified at various time points following crizotinib treatment of H3122 cells. Red dots represent genes significantly upregulated and blue dots represent genes significantly downregulated. (B) Table summarizes the significantly enriched pathways commonly identified in all treatment stages of crizotinib-treated H3122 cells.

## Slide 2
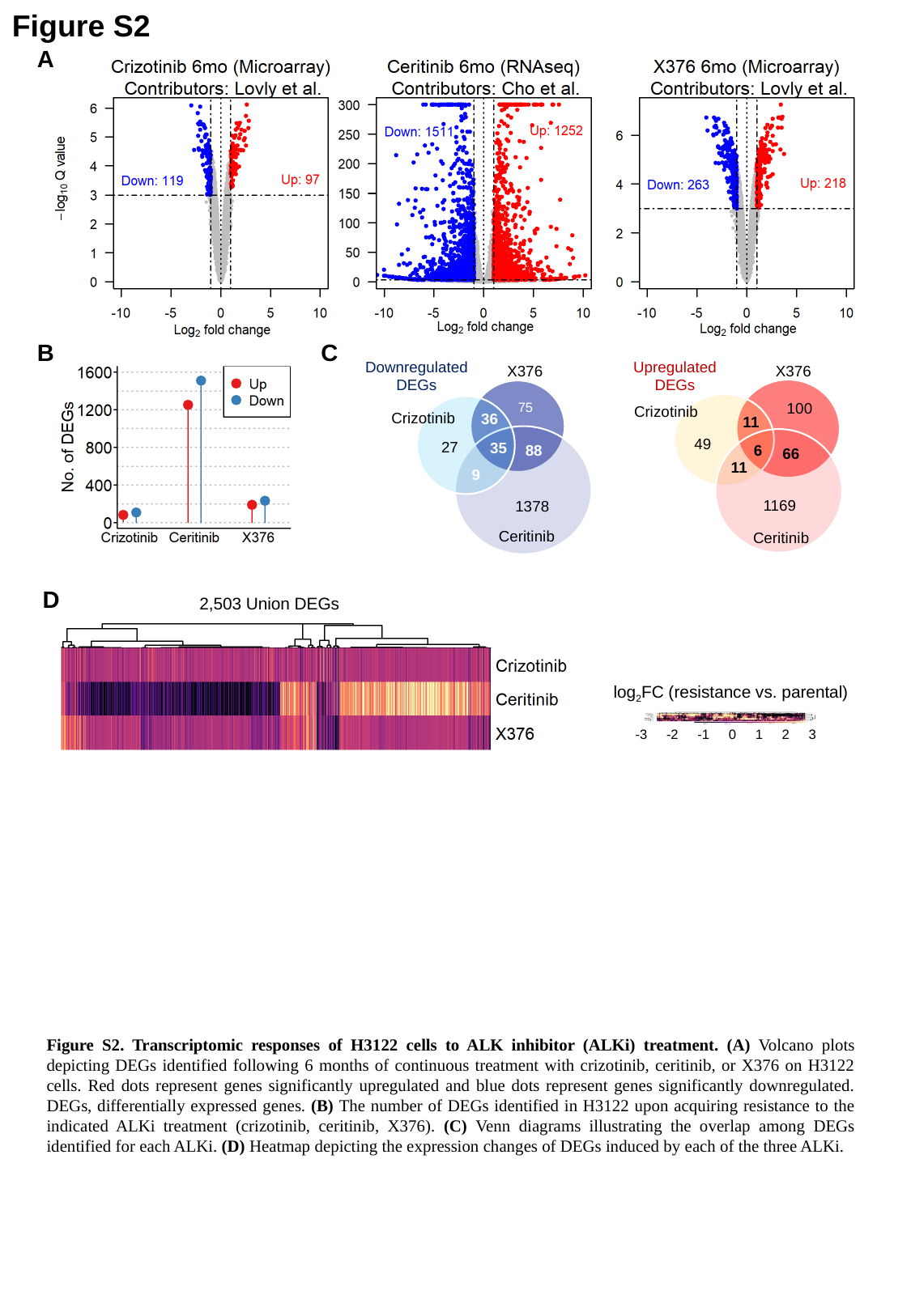

Figure S2
A
B
C
Downregulated
DEGs
X376
75
Crizotinib
36
27
35
88
9
1378
Ceritinib
Upregulated DEGs
X376
100
Crizotinib
11
49
6
66
11
1169
Ceritinib
D
2,503 Union DEGs
log2FC (resistance vs. parental)
-3 -2 -1 0 1 2 3
Figure S2. Transcriptomic responses of H3122 cells to ALK inhibitor (ALKi) treatment. (A) Volcano plots depicting DEGs identified following 6 months of continuous treatment with crizotinib, ceritinib, or X376 on H3122 cells. Red dots represent genes significantly upregulated and blue dots represent genes significantly downregulated. DEGs, differentially expressed genes. (B) The number of DEGs identified in H3122 upon acquiring resistance to the indicated ALKi treatment (crizotinib, ceritinib, X376). (C) Venn diagrams illustrating the overlap among DEGs identified for each ALKi. (D) Heatmap depicting the expression changes of DEGs induced by each of the three ALKi.

## Slide 3
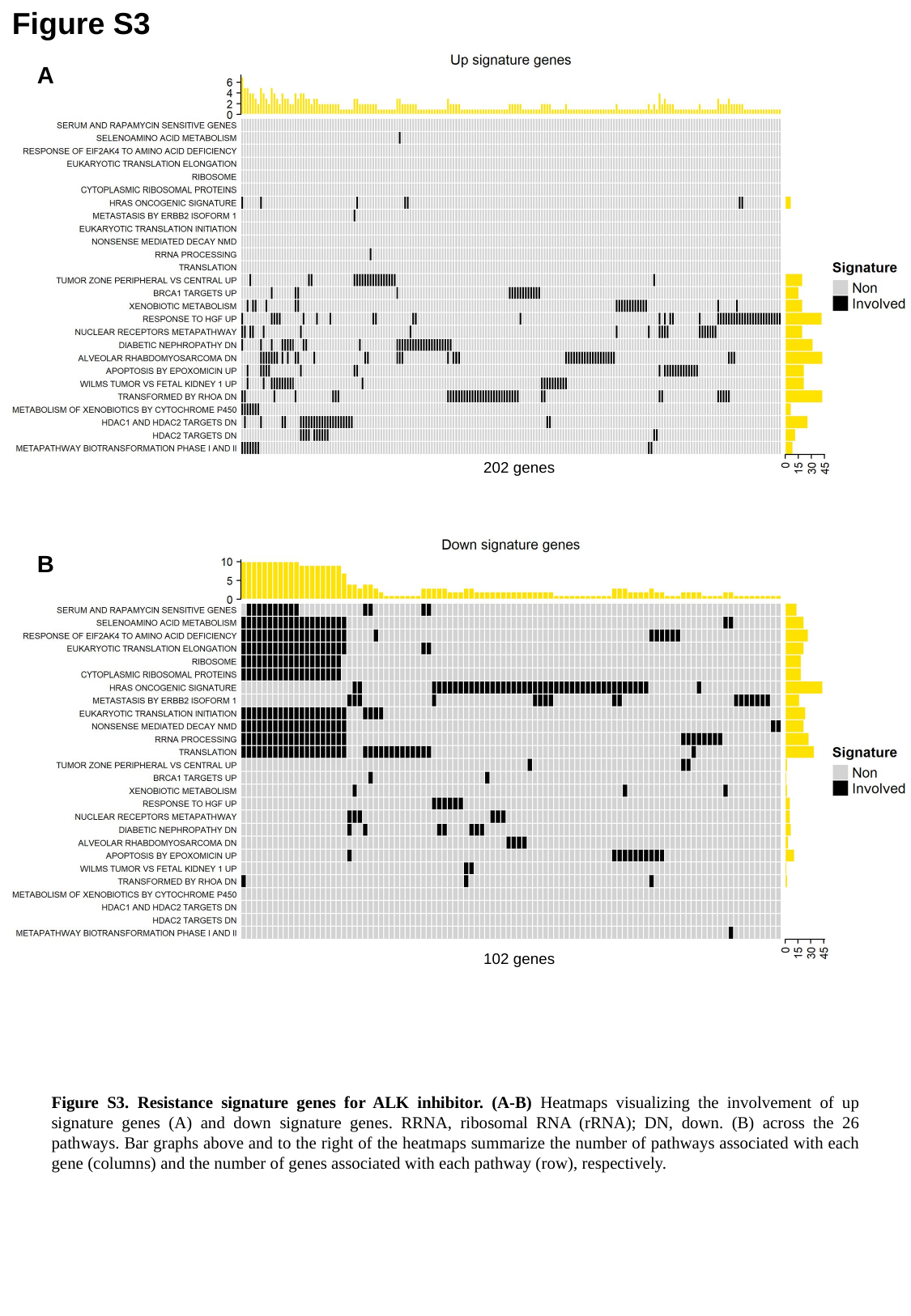

Figure S3
A
202 genes
B
102 genes
Figure S3. Resistance signature genes for ALK inhibitor. (A-B) Heatmaps visualizing the involvement of up signature genes (A) and down signature genes. RRNA, ribosomal RNA (rRNA); DN, down. (B) across the 26 pathways. Bar graphs above and to the right of the heatmaps summarize the number of pathways associated with each gene (columns) and the number of genes associated with each pathway (row), respectively.

## Slide 4
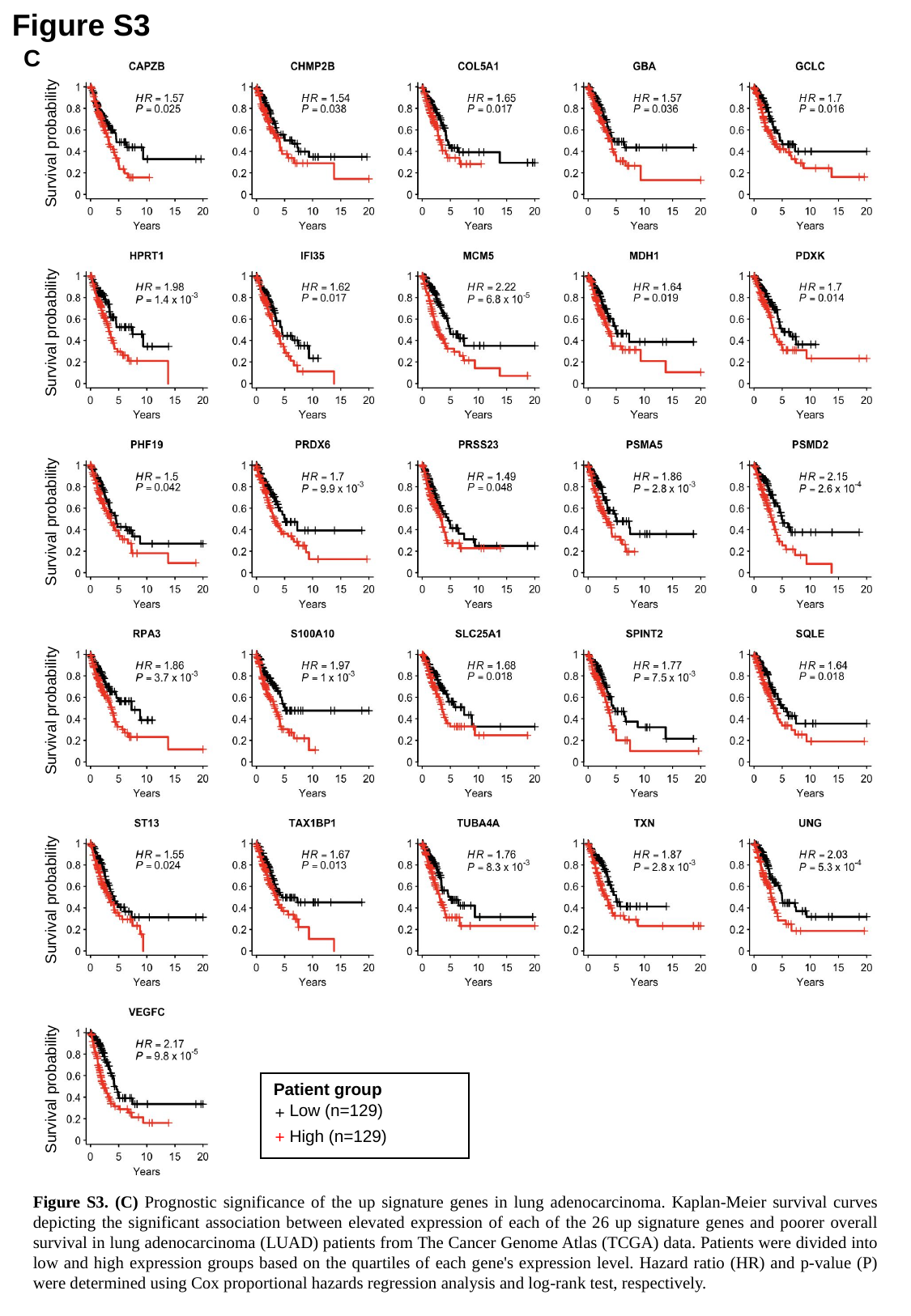

Figure S3
C
Survival probability
Survival probability
Survival probability
Survival probability
Survival probability
Survival probability
Patient group
Low (n=129)
+
High (n=129)
+
Figure S3. (C) Prognostic significance of the up signature genes in lung adenocarcinoma. Kaplan-Meier survival curves depicting the significant association between elevated expression of each of the 26 up signature genes and poorer overall survival in lung adenocarcinoma (LUAD) patients from The Cancer Genome Atlas (TCGA) data. Patients were divided into low and high expression groups based on the quartiles of each gene's expression level. Hazard ratio (HR) and p-value (P) were determined using Cox proportional hazards regression analysis and log-rank test, respectively.

## Slide 5
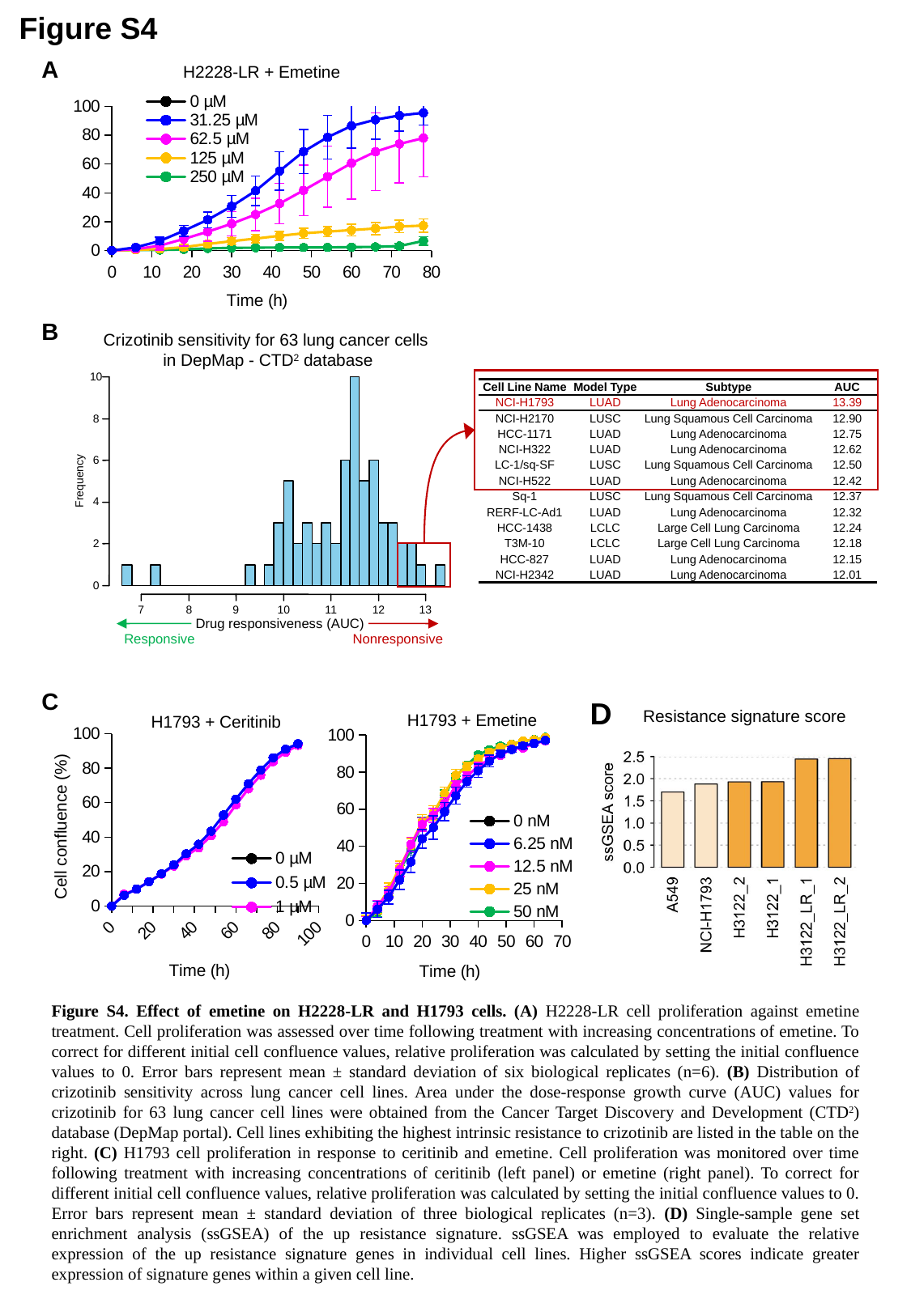

Figure S4
A
H2228-LR + Emetine
### Chart
| Category | 0 µM | 31.25 µM | 62.5 µM | 125 µM | 250 µM |
|---|---|---|---|---|---|B
Crizotinib sensitivity for 63 lung cancer cells
 in DepMap - CTD2 database
10
8
6
Frequency
4
2
0
7
8
9
10
11
12
13
Drug responsiveness (AUC)
Responsive
Nonresponsive
| Cell Line Name | Model Type | Subtype | AUC |
| --- | --- | --- | --- |
| NCI-H1793 | LUAD | Lung Adenocarcinoma | 13.39 |
| NCI-H2170 | LUSC | Lung Squamous Cell Carcinoma | 12.90 |
| HCC-1171 | LUAD | Lung Adenocarcinoma | 12.75 |
| NCI-H322 | LUAD | Lung Adenocarcinoma | 12.62 |
| LC-1/sq-SF | LUSC | Lung Squamous Cell Carcinoma | 12.50 |
| NCI-H522 | LUAD | Lung Adenocarcinoma | 12.42 |
| Sq-1 | LUSC | Lung Squamous Cell Carcinoma | 12.37 |
| RERF-LC-Ad1 | LUAD | Lung Adenocarcinoma | 12.32 |
| HCC-1438 | LCLC | Large Cell Lung Carcinoma | 12.24 |
| T3M-10 | LCLC | Large Cell Lung Carcinoma | 12.18 |
| HCC-827 | LUAD | Lung Adenocarcinoma | 12.15 |
| NCI-H2342 | LUAD | Lung Adenocarcinoma | 12.01 |
C
D
H1793 + Emetine
H1793 + Ceritinib
Resistance signature score
### Chart
| Category | 0 µM | 0.5 µM | 1 µM |
|---|---|---|---|
### Chart
| Category | 0 nM | 6.25 nM | 12.5 nM | 25 nM | 50 nM |
|---|---|---|---|---|---|
Cell confluence (%)
Figure S4. Effect of emetine on H2228-LR and H1793 cells. (A) H2228-LR cell proliferation against emetine treatment. Cell proliferation was assessed over time following treatment with increasing concentrations of emetine. To correct for different initial cell confluence values, relative proliferation was calculated by setting the initial confluence values to 0. Error bars represent mean ± standard deviation of six biological replicates (n=6). (B) Distribution of crizotinib sensitivity across lung cancer cell lines. Area under the dose-response growth curve (AUC) values for crizotinib for 63 lung cancer cell lines were obtained from the Cancer Target Discovery and Development (CTD2) database (DepMap portal). Cell lines exhibiting the highest intrinsic resistance to crizotinib are listed in the table on the right. (C) H1793 cell proliferation in response to ceritinib and emetine. Cell proliferation was monitored over time following treatment with increasing concentrations of ceritinib (left panel) or emetine (right panel). To correct for different initial cell confluence values, relative proliferation was calculated by setting the initial confluence values to 0. Error bars represent mean ± standard deviation of three biological replicates (n=3). (D) Single-sample gene set enrichment analysis (ssGSEA) of the up resistance signature. ssGSEA was employed to evaluate the relative expression of the up resistance signature genes in individual cell lines. Higher ssGSEA scores indicate greater expression of signature genes within a given cell line.

## Slide 6
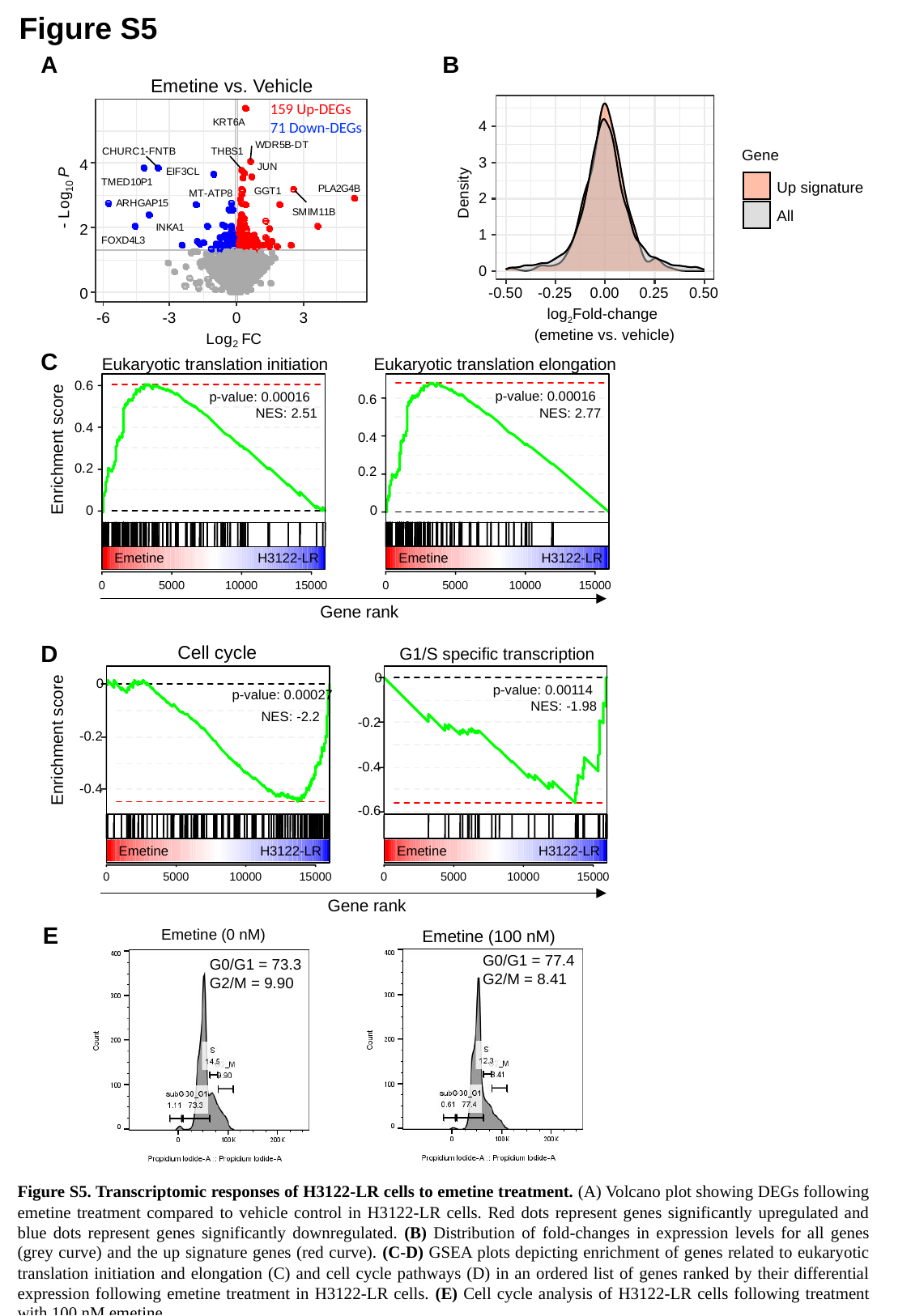

Figure S5
A
B
Emetine vs. Vehicle
Gene
Up signature
Density
All
log2Fold-change
(emetine vs. vehicle)
159 Up-DEGs
71 Down-DEGs
C
Eukaryotic translation elongation
p-value: 0.00016
0.6
NES: 2.77
0.4
0.2
0
Emetine
H3122-LR
0
5000
10000
15000
Eukaryotic translation initiation
0.6
p-value: 0.00016
NES: 2.51
0.4
Enrichment score
0.2
0
Emetine
H3122-LR
0
5000
10000
15000
Gene rank
D
Cell cycle
0
p-value: 0.00027
NES: -2.2
-0.2
Enrichment score
-0.4
Emetine
H3122-LR
0
5000
10000
15000
G1/S specific transcription
0
p-value: 0.00114
NES: -1.98
-0.2
-0.4
-0.6
Emetine
H3122-LR
0
5000
10000
15000
Gene rank
E
Emetine (0 nM)
Emetine (100 nM)
G0/G1 = 77.4
G2/M = 8.41
G0/G1 = 73.3
G2/M = 9.90
Figure S5. Transcriptomic responses of H3122-LR cells to emetine treatment. (A) Volcano plot showing DEGs following emetine treatment compared to vehicle control in H3122-LR cells. Red dots represent genes significantly upregulated and blue dots represent genes significantly downregulated. (B) Distribution of fold-changes in expression levels for all genes (grey curve) and the up signature genes (red curve). (C-D) GSEA plots depicting enrichment of genes related to eukaryotic translation initiation and elongation (C) and cell cycle pathways (D) in an ordered list of genes ranked by their differential expression following emetine treatment in H3122-LR cells. (E) Cell cycle analysis of H3122-LR cells following treatment with 100 nM emetine.
