# Supplementary material for: Integrative transcriptomic analysis identifies emetine as a promising candidate for overcoming acquired resistance to ALK inhibitors in lung cancer
Source: Mol Oncol. 2024 Nov 14;19(4):1155–69. doi: 10.1002/1878-0261.13738 (PMC11977641; doi:10.1002/1878-0261.13738)
Supplement: Supplementary file 2 — Table S1. Public transcriptome datasets derived from H3122 cell lines with different durations of crizotinib treatment. Table S2. Public transcriptome datasets derived from H3122 cell lines with ceritinib or X376 treatment. [file MOL2-19-1155-s001.pdf]

Table S1. Public transcriptome datasets derived from H3122 cell lines with different durations of crizotinib treatment.

GEO ID #	Sample ID #	Sample Labels	Cell type	Group	Group #
GSE62663: Fasting potentiates the anticancer activity of Crizotinib Organism [Microarray]	GSM1531063	Control, replicate 1	H3122	Control	Crizotinib 72h vs Control
	GSM1531064	Control, replicate 2	H3122	Control	
	GSM1531065	Control, replicate 3	H3122	Control	
	GSM1531066	Control, replicate 4	H3122	Control	
	GSM1531067	Crizotinib, replicate 1	H3122	Crizotinib 72h	
	GSM1531068	Crizotinib, replicate 2	H3122	Crizotinib 72h	
	GSM1531069	Crizotinib, replicate 3	H3122	Crizotinib 72h	
	GSM1531070	Crizotinib, replicate 4	H3122	Crizotinib 72h	
GSE89127: Suppression of adaptive responses to targeted cancer therapy by transcriptional repression [RNA-seq]	GSM2359221	H3122-Cr2 Criz	H3122	Crizotinib 48h	Crizotinib 48h vs Control
	GSM2359222	H3122-Cr3 Criz	H3122	Crizotinib 48h	
	GSM2359223	H3122-Cr4 Criz	H3122	Crizotinib 48h	
	GSM2359224	H3122-D2 DMSO	H3122	Control	
	GSM2359225	H3122-D3 DMSO	H3122	Control	
	GSM2359226	H3122-D4 DMSO	H3122	Control	
	GSM2359227	H3122-H1 Criz_7days	H3122	Crizotinib 7d	Crizotinib 7days vs Control
	GSM2359228	H3122-H3 Criz_7days	H3122	Crizotinib 7d	
	GSM2359229	H3122-H4 Criz_7days	H3122	Crizotinib 7d	
GSE49508: Analysis of ALK TKI sensitive and ALK TKI resistant isogenic pairs of H3122 (EML4-ALK E13;A20) cells [Microarray]	GSM1200187	sensitive to PF1066 (aka crizotinib) and X-376	H3122	Parental	Crizotinib 6M vs Control
	GSM1200188	sensitive to PF1066 (aka crizotinib) and X-376	H3122	Parental	
	GSM1200189	sensitive to PF1066 (aka crizotinib) and X-376	H3122	Parental	
	GSM1200190	resistant to the ALK TKI, PF1066 (aka crizotinib)	H3122	Crizotinib-Resistant	
	GSM1200191	resistant to the ALK TKI, PF1066 (aka crizotinib)	H3122	Crizotinib-Resistant	
	GSM1200192	resistant to the ALK TKI, PF1066 (aka crizotinib)	H3122	Crizotinib-Resistant	

Table S2. Public transcriptome datasets derived from H3122 cell lines with ceritinib or X376 treatment.

GEO ID #	Sample ID #	Sample Labels	Cell type	Group	Group #
GSE49508: Analysis of ALK TKI sensitive and ALK TKI resistant isogenic pairs of H3122 (EML4-ALK E13;A20) cells [Microarray]	GSM1200187	sensitive to PF1066 (aka crizotinib) and X-376	H3122	Parental	X376 6M vs Control
	GSM1200188	sensitive to PF1066 (aka crizotinib) and X-376	H3122	Parental	
	GSM1200189	sensitive to PF1066 (aka crizotinib) and X-376	H3122	Parental	
	GSM1200193	resistant to the ALK TKI, X-376	H3122	X376-Resistant	
	GSM1200194	resistant to the ALK TKI, X-376	H3122	X376-Resistant	
	GSM1200195	resistant to the ALK TKI, X-376	H3122	X376-Resistant	
GSE81484: Gene expression profiling study by RNA-seq for identifying genes associated with epithelial-mesenchymal transition and acquired resistance to ALK inhibitors [RNA-seq]	GSM2154748	H3122_1_RNA-seq	H3122	Parental	Ceritinib 6M vs Control
	GSM2154749	H3122_LR_1_RNA-seq	H3122	Ceritinib-Resistant	
	GSM2154751	H3122_2_RNA-seq	H3122	Parental	
	GSM2154752	H3122_LR_2_RNA-seq	H3122	Ceritinib-Resistant	
